# Multi-omics analyses identify EZH2 as a central driver in rhabdomyosarcoma radioresistance and highlight Tazemetostat as an effective radiosensitizer in vitro and in vivo

**DOI:** 10.1038/s41419-026-08938-0

**Published:** 2026-06-01

**Authors:** Matteo Cassandri, Antonella Porrazzo, Simona Camero, Silvia Pomella, Valeria Manzi, Francesca Vulcano, Luisa Milazzo, Francesca Pedini, Deborah Pajalunga, Andrea Casagrande, Giada Mele, Valentina Lulli, Enrico Romano, Michele Signore, Massimo Spada, Maria Teresa D’Urso, Silvia Codenotti, Alessandro Fanzani, Annunziata Mauro, Giovanna Marchese, Simone Sidoli, Giovanni Luca Gravina, Giovanni Barillari, Franco Locatelli, Francesca Megiorni, Rossella Rota, Francesco Marampon

**Affiliations:** 1 Department of Experimental Medicine, “Sapienza” University of Rome, Rome, Italy; 2 Department of Hematology/Oncology, Cell and Gene Therapy, Bambino Gesù Children’s Hospital, IRCCS, Rome, Italy; 3 Department of Radiological, Oncological and Pathological Sciences, “Sapienza” University of Rome, Policlinico Umberto I, Rome, Italy; 4 Department of Life Sciences, Health and Health Professions, Link Campus University, Rome, Italy; 5 Department of Clinical Sciences and Translational Medicine, University of Rome Tor Vergata, Rome, Italy; 6 Department of Oncology and Molecular Medicine, Istituto Superiore di Sanità, Rome, Italy; 7 Department of Sense Organs, Sapienza University of Rome, Rome, Italy; 8 RPPA Unit, Proteomics Area, Core Facilities, Istituto Superiore di Sanità, Rome, Italy; 9 Center of Animal Research and Welfare, Istituto Superiore di Sanità, Rome, Italy; 10 Department of Molecular and Translational Medicine, University of Brescia, Brescia, Italy; 11 Faculty of Bioscience and Technology for Food, Agriculture and Environment, University of Teramo, Teramo, Italy; 12 Genomix4Life S.r.l, Baronissi, Italy; 13 Genome Research Center for Health, CRGS, Baronissi, Italy; 14 Department of Biochemistry, Albert Einstein College of Medicine, Bronx, NY USA; 15 Department of Biotechnological and Applied Clinical Sciences, Radiation Oncology Unit, Ospedale San Salvatore, University of L’Aquila, L’Aquila, Italy; 16 Department of Life Sciences and Public Health, Catholic University of the Sacred Heart, Rome, Italy; 17 Department of Well-being, Health and Environmental Sustainability, Sapienza University of Rome, Rieti, Italy

**Keywords:** Sarcoma, Proteomics, Transcriptomics

## Abstract

Rhabdomyosarcoma (RMS), a pediatric soft tissue sarcoma, comprises two major subtypes: fusion-positive (FP), driven by PAX3/7-FOXO1 fusions, and fusion-negative (FN), often harboring RAS pathway mutations. High-risk RMS exhibits intrinsic resistance to radiotherapy (RT), posing a significant clinical hurdle. Emerging evidence implicates EZH2, the catalytic subunit of the Polycomb Repressive Complex 2 (PRC2), in promoting RT resistance through gene silencing via H3K27me3. To dissect the molecular basis of RMS radioresistance, we employed an integrative multi-omics approach encompassing phosphoproteomics and transcriptomics. Radioresistant RMS cell models (RMS^CRR^) displayed enhanced cancer stem cell features, evasion of RT-induced G2/M arrest, and reduced apoptosis compared to their parental counterparts (RMS^PR^). Phosphoproteomic profiling revealed activation of prosurvival and proliferative pathways across both FN and FP subtypes. Transcriptomic analysis identified a robust downregulation of EZH2 target genes, with distinct gene sets modulated in FN-RMS^CRR^ *versus* FP-RMS^CRR^ cells, highlighting subtype-specific epigenetic rewiring. These multi-omics findings pointed to hyperactive PRC2/EZH2 signaling as a driver of radioresistance. Therapeutically, combining the EZH2 inhibitor Tazemetostat (TZM) with RT significantly impaired clonogenic survival, enhanced G2/M arrest, and promoted apoptosis in both RMS^PR^ and RMS^CRR^ cells. In vivo, RT and TZM co-treatment fully suppressed FN-RMS^PR^ tumor growth and delayed FP-RMS^PR^ progression. Notably, TZM monotherapy inhibited tumor growth in both FN- and FP-RMS^CRR^ xenografts, uncovering a therapy-induced vulnerability. Our integrative multi-omics analysis reveals EZH2-dependent molecular programs underpinning radioresistance and supports EZH2 targeting as a rational radiosensitizing and therapeutic strategy in RMS, including recurrent and RT-refractory disease.

## Introduction

Rhabdomyosarcoma (RMS) is the most common type of soft tissue sarcoma (STS) that originates from myogenic precursors [1, 2]. Histologically, RMS includes two main subtypes: alveolar (ARMS) and embryonal (ERMS) [1–3]. About 80% of ARMS carries chromosomal translocations that lead to the formation of the oncogenic fusion proteins PAX3-FOXO1 (P3F) or PAX7-FOXO1 [1–3]. ERMS lacks any fusions (fusion-negative (FN)-RMS) but frequently exhibits mutations of RAS pathway and/or a limited set of other signaling pathways [1–3]. Interestingly, many of these involves PAX3/7-FOXO1 targets suggesting that FP-RMS can share overlapping molecular mechanisms with FN-RMS [1]. FP-RMS and metastatic/recurrent FN-RMS are high-risk (HR) entities with < 30% of 5-year overall survival [4]. The management of HR RMS patients mainly requires combining surgery, radiotherapy (RT), and multi-agent systemic chemotherapy to eradicate disseminated cancer cells [5]. RT is critical for the local control of the disease [6]. Unfortunately, despite RT dose escalation and the use of hypofractionated RT, i.e., larger dose of radiation *per* single fraction [7], local failure after RT is the predominant type of relapse, particularly in HR RMS patients [8]. Thus, the identification of molecular mechanisms responsible for the intrinsic and/or acquired radioresistance in HR RMS is urgently needed to enhance RT efficacy [9, 10].

In this view, cancer cells surviving to clinically used dose of RT, i.e., clinically relevant radioresistant (CRR) cells, are considered preferred models to study radioresistance as compared to their isogenic background, representing post-irradiation radioresistant recurrent cells [11–18]. Accordingly, we have recently developed and functionally characterized as radioresistant one FN- and one FP-RMS^CRR^ cell line showing that both survive to a clinically used hypofractionated schedule of RT [18].

Here, to comprehensively evaluate the molecular signatures of RMS^CRR^ cells to identify common orchestrators that can be therapeutically targeted to overcome radioresistance, we expanded the radioresistant models by developing two additional cell lines, both FN- and FP-RMS^CRR^. An integrated analysis of transcriptomic and phosphoproteomic/proteomic profiles of RMS^CRR^ *versus* RMS^PR^ cell lines indicate activation of survival pathways. Moreover, the Polycomb Repressive Complex 2 (PRC2)/EZH2, which represses gene expression through the trimethylation of histone H3 on lysine 27 (H3K27me3), known to be recurrently mutated in several forms of cancer or highly expressed in numerous others [19], and to sustain radioresistance in different cancer types [20–25], was shown hyperactivated in RMS^CRR^ by transcriptomic profile analysis. In agreement, EZH2 pharmacological inhibition with the investigational inhibitor Tazemetostat (TZM) reduced colony formation efficiency and DNA repair and increased apoptosis in vitro when combined with RT. Moreover, TZM administered with RT in vivo strongly radiosensitized FN-RMS^PR^ cells, and partially inhibited the intrinsically pharmaco- and radioresistant FP-RMS^PR^ compared to each single treatment. Finally, TZM as a single treatment was able to prevent FN-RMS^CRR^ tumor growth and remarkably impair that of FN-RMS^CRR^ cells unveiling an acquired vulnerability to EZH2 inhibition that could be therapeutically targeted.

## Materials and methods

### Cell lines, tumorsphere culture, and pharmacological treatment

RD, SMS-CTR (FN-RMS) and RH30, RH4 (FP-RMS) human cell lines were purchased from American Type Culture Collection (Manassas, VA, USA). JR1 (FN-RMS) was gifted by Dr. Peter Houghton, and SCMC (FP-RMS) by Dr. Janet Shipley. Cells were maintained as already described [26]. Cell line authentication was performed using the GenePrint 10 System (Promega Corporation, Madison, WI, USA). Sphere-forming cells were obtained, and radiation treatment was performed as already described [26]. One day after plating, cells were treated with Tazemetostat (TZM) purchased from Selleckchem (Houston, TX, USA). Countess II Automated Cell Counter (ThermoFisher Scientific, Waltham, MA, USA) was used to assess the number of cells.

### Cell cycle and apoptosis analysis by Flow cytometry

For cell cycle analysis, cells were fixed in a cold solution of 50% PBS, 5% FBS and 50% acetone/methanol (1:4 v/v) over night at +4 °C. Cells were then treated with RNase A (Sigma-Aldrich, St Louis, MO, USA) and stained with Propidium Iodide (PI) (ThermoFisher Scientific, Rockford, USA), as previously described [27]. BD FACSCalibur (BD Biosciences, Franklin Lakes, NJ, USA), equipped with ModFit LT 3.0 program (Verity Software House), was used to acquire, and quantify flow cytometry data [26]. For apoptosis analysis, 1.5 × 10^6^ cells were seeded, irradiated and after 24 h they were incubated with Annexin V and 7-Aminoactinomycin D (7-AAD), using FITC Annexin V apoptosis detection kit (BD Pharmingen, San Diego, CA, USA), according to manufacturer’s recommendations. Cells were analyzed using FACS CantoII equipped with a FACSDiva 6.1 CellQuest software (Becton Dickinson Instrument, San Josè, CA, USA).

### Radiation exposure and clonogenic assay

Radiation was delivered at room temperature using a x-6 MV photon linear accelerator, at a dose rate of 2 Gray/minute (Gy/min) using a source-to-surface distance (SSD) of 100 cm as already described [28, 29]. For clonogenic survival assay, exponentially growing RMS cells were treated with TZM or vehicle and irradiated 24 hours (h) later. Three hours after irradiation, cells were harvested, counted, serially diluted to appropriate densities, plated in triplicate in 6-well plates with 2 ml of complete drug-free medium/each well. Fourteen days later, cells were fixed with methanol:acetic acid (10:1, v/v) and stained with crystal violet. Colonies containing >50 cells were counted [26].

### RNA knocking down by small interfering RNAs

RD^PR^ cells and RH30^PR^ were transiently transfected with small interfering RNAs (siRNAs) as previously described [28]. Specifically, siRNAs against human MTSS1 (si-MSTT1: sc-77651 by Santa Cruz Biotechnology, Dallas, TX, USA) and HHIP (si-HHIP: sc-43835 by Santa Cruz Biotechnology, Dallas, TX, USA) were used in RD^PR^ cells and in RH30^PR^, respectively. A negative control siRNA (si-NC: sc-37007 by Santa Cruz Biotechnology, Dallas, TX, USA) served for evaluating RNA interference’s off-target effects. Forty-eight hours after transfection, cells were irradiated at a single dose of 6 Gy and then used at different time points for subsequent specific assays.

### Protein extraction, immunoblotting, and capillary western blot analysis

For total protein extraction, cells were lysed in 2% SDS containing 2 mM PMSF, 10 μg/ml antipain, leupeptin, and trypsin inhibitor, 10 mM sodium fluoride and 1 mM sodium orthovanadate. Immunoblotting experiments were performed as previously described [30]. Protein Simple WES instrument (ProteinSimple, San Jose, CA, USA) was used for quantitative capillary electrophoresis-based technique for western analysis as already described [31]. For primary antibody, phospho-ATM (D6H9), phospho-DNAPKcs (E9J4G), DNAPKcs (E6U3A) and MTSS1 (D2H4L) by Cell Signaling (Danvers, MA, USA); ATM (G-12); c-Myc (9E10), N-Myc (B.8.4.B), phospho-ERK1/2 (E-4), ERK1/2 (C-14), phospho-Akt (C-11), Akt (5C10), γH2AX (938CT5.1), RAD51 (F-11), ku70 (A-9), MyoD (4H207) and HHIP (5D11) by Santa Cruz Biotechnology (Dallas, TX, USA); Vinculin (hVIN-1) by Sigma-Aldrich (Saint Louis, MO, USA). Anti-mouse HRP and anti-rabbit HRP secondary antibodies from ProteinSimple were used [31].

### RNA-seq sample preparation and data analysis

RNA was extracted from RD, SMS-CTR, RH30, and RH4 (CRR and PR) using the RNeasy mini kit (#74104, Qiagen, Hilden, Germany) according to the manufacturer’s instructions. Poly-A selected RNA libraries were prepared using the NEBNext Ultra II Directional RNA Library Prep kit and sequenced on an Illumina NovaSeq6000 System (2 × 150 bp). Reads were aligned to the GRCh38 reference genome using STAR version 2.7.10b, and the quantification of transcripts expressed for each sequenced sample was performed using featureCount algorithm (version 2.0.3). Gene Set Enrichment Analysis (GSEA, https://www.gsea-msigdb.org/gsea/index.jsp) was performed considering log2FC > 1.5 for upregulated genes and a log2FC < −1.5 for downregulated genes. Bubble-plots of enriched output from GSEA analysis were created in R using custom scripts [32].

### Bioinformatic analysis

Expression profiles of EZH2, SUZ12, and EED in RMS biopsies and in normal skeletal muscle were obtained using Williamson dataset (E-TABM-1202) and Asman dataset (GSE9103) available at R2 Genomics Analysis and Visualization Platform (https://hgserver1.amc.nl/cgi-bin/r2/main.cgi) [32]. Data from CRISPR genome screening on 11 RMS cell lines were downloaded from DepMap (https://depmap.org/portal/). Perturbation gene effects were reported as Chronos score. The association between expression levels of EZH2, SUZ12, and EED and the overall survival of RMS patients was obtained using the Williamson dataset (E-TABM-1202). *P* values were determined by log rank test, where the data were dichotomized into lowly expressed and highly expressed groups by percentiles. The best separation, smallest *p* value was then reported, accompanied by a Kaplan Meier picture. All data were plotted using GraphPad Prism 9. Chromatin Immunoprecipitation (ChIP)-seq data for EZH2 in SMS-CTR were retrieved from Gene Expression Omnibus dataset (GEO dataset) under the accession code GSE85171 and visualized with Integrated Genome Viewer (IGV) version 2.19.1.

### ChIP analysis

RD^PR^, RD^RR^, RH30^PR^, and RH30^PR^ (2 × 10^6^ cells) were crosslinked for 10 min in a solution of 1% formaldehyde, and processed for ChIP assays using the MAGnify™ Chromatin Immunoprecipitation System (Invitrogen Cat. 492024), according to manufacturer’s instructions. Briefly, cells were lysed and then sonicated to obtain chromatin fragments of ~200–500 bp. The lysates were immunoprecipitated using specific antibodies, anti-EZH2 (CST #5246) and anti-H3K27me3 (CST #9733) and non-specific IgG as negative control. DNA fragments were analyzed by qRT-PCR using specific primers (MTSS1 Peak_7938: FW CCTCCATCTGGGAAGAACCA, REV GTGATGAGTCAGCAGCCACT; HHIP Peak_6427: FW AACAGAGTCTGCTGATCCCA, REV GGAACTGCCTGTTGCAGATG).

### Reverse phase protein array

1.5 × 10^6^ cells were seeded and treated with 6 Gy of irradiation after 24 h. Cells were harvested at 10 min and 3 h post-RT. Protein extracts were diluted and spotted onto nitrocellulose-coated slides. These extracts were arranged in a 4 × 12-pin-11 × 11 subarray that accommodate 1056 serially diluted samples plus replicate vertical controls on each slide. The protein of interest was detected by probing with a specific antibody, amplifying the signal via a tyramide amplification system, and visualized via DAB colorimetric reaction. The detection system used was a GenPoint-based staining kit from Agilent. Digital images of slides were obtained by scanning on a Huron TissueScope scanner producing 16-bit TIFF files. Mean net intensities of spots from all 1056 samples were used for curve-fitting for each slide (RPPA Super Position and Concentration Evaluation, aka SPACE). Relative protein level for each sample was determined by converging its sample dilution series to one point (EC50) and interpolating in RPPASPACE. All the procedures have been performed as service at the Functional Proteomics RPPA Core of MD Anderson Cancer Center (Houston, TX, USA). Data were plotted as Principal Component Analysis and Heatmap using SRplot [32].

### S-trap protein digestion

Samples were homogenized with a pestle and probe sonicator in a buffer containing 5% SDS, 5 mM DTT and 50 mM ammonium bicarbonate (pH = 8) and left on the bench for about 1 h for disulfide bond reduction. Samples were then alkylated with 20 mM iodoacetamide in the dark for 30 min. Afterward, phosphoric acid was added to the sample at a final concentration of 1.2%. Samples were diluted in 6 volumes of binding buffer (90% methanol and 10 mM ammonium bicarbonate, pH 8.0). After gentle mixing, the protein solution was loaded to an S-trap filter (ProtiFi, Fairport NY, USA) and spun at 500 × *g* for 30 s. The sample was washed twice with the binding buffer. Finally, 1 µg of sequencing grade trypsin (Promega, Madison, WI, USA), diluted in 50 mM ammonium bicarbonate, was added into the S-trap filter and samples were digested at 37 °C for 18 h. Peptides were eluted in three steps: (i) 40 µl of 50 mM ammonium bicarbonate, (ii) 40 µl of 0.1% TFA and (iii) 40 µl of 60% acetonitrile and 0.1% TFA. Prior to mass spectrometry analysis, samples were desalted using a 96-well plate filter (Orochem, Naperville, IL, USA) packed with 1 mg of Oasis HLB C-18 resin (Waters, Milford MA, USA).

### LC-MS/MS acquisition and analysis

Samples were resuspended in 10 µl of 0.1% TFA and loaded onto a Dionex RSLC Ultimate 300 (Thermo Scientific), coupled online with an Orbitrap Fusion Lumos (ThermoFisher Scientific, Waltham, MA, USA). Chromatographic separation was performed with a two-column system, consisting of a C-18 trap cartridge (300 µm ID, 5 mm length) and a picofrit analytical column (75 µm ID, 25 cm length) packed in-house with reversed-phase Repro-Sil Pur C18-AQ 3 µm resin. Peptides were separated using a 90 min gradient from 4 to 30% buffer B (buffer A: 0.1% formic acid, buffer B: 80% acetonitrile + 0.1% formic acid) at a flow rate of 300 nl/min. The mass spectrometer was set to acquire spectra in a data-dependent acquisition (DDA) mode. Proteome raw files were searched using Proteome Discoverer software (v2.5, ThermoFisher Scientific, Waltham, MA, USA) using SEQUEST search engine and the SwissProt human database. Mass tolerance was set to 10 pm for precursor ions and 0.2 Da for product ions. The peptide and protein false discovery rate was set to 1%. Statistical regulation was assessed using heteroscedastic T-test (if *p*-value < 0.05).

### In vivo xenograft and radiation exposure

Female nude CD1 mice were provided by Charles River Laboratories (Calco, Italy), aged 6–8 weeks and used for xenografts experiments. Animals were maintained in sterile conditions, with a cycle of 12 h light/12 h dark, 18–23 °C ambient temperature and 40–60% humidity. Before any invasive manipulation, mice were anesthetized with a mixture of ketamine (25 mg/ml)/xylazine (5 mg/ml). RMS^PR^ and RMS^CRR^, 5 × 10^6^ each, were subcutaneously injected in the left flank of the mice. When the masses were palpable, tumor sizes were measured, in a blinding manner, weekly throughout the treatment period and for the following two weeks by caliper. Experimental mice were randomized into the experimental groups following tumor inoculation treatments. TZM (HY-13803, MedChem Express, Monmouth Junction, NJ, USA) or vehicle was administered intraperitoneally twice a day for 5 days per week for 4 weeks at the dose of 75 mg/Kg. For the in vivo RT model, mice were irradiated as already described [33]. Mouse experiments were conducted in compliance with the international, EU and national ethical requirements and were approved by the Italian Health Ministry 221/2022-PR (D9997.140).

### Statistical analysis

Statistical analyses were performed using GraphPad Prism 10.1.1. Differences among multiple groups were assessed by one-way ANOVA followed by appropriate multiple-comparison tests, while single comparisons were evaluated using Student’s t-test. A *p* value < 0.05 was considered statistically significant, and significant values are indicated in the figures. The sample size for animal studies was determined under the assumption of minimal variability within groups, following the principle of using the lowest number of animals necessary. Calculations were based on detecting a difference of two standard deviations with 80% power at a 0.05 significance level. No formal sample size calculation was conducted for in vitro experiments; however, each experimental condition was performed with three biological replicates, in line with standard practice to account for expected biological variability.

## Results

### RMS surviving to ultra-hypofractionated RT increases the content of radioresistant CSCs-like cells

**Fig. 1:**
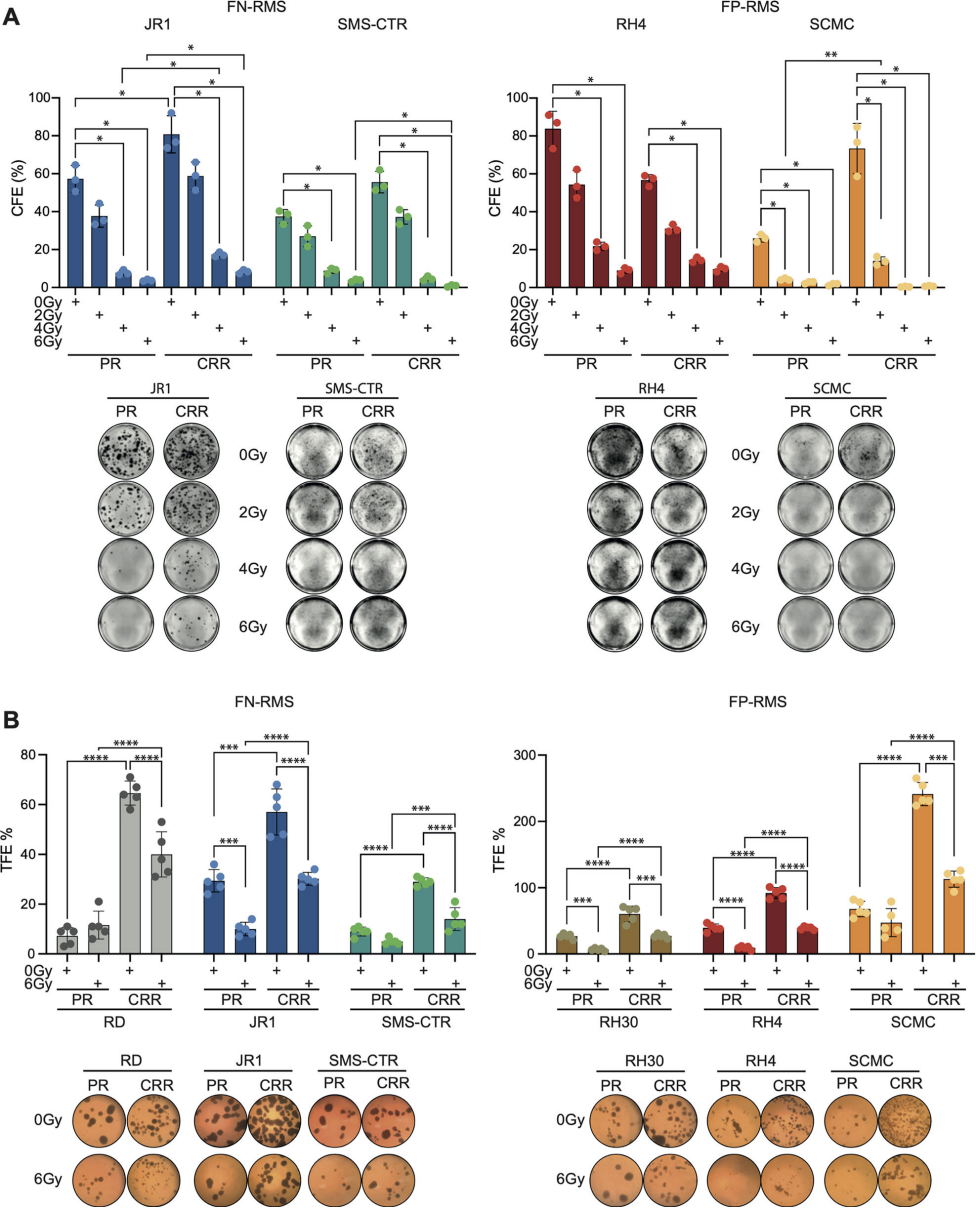
RT survived RMS cell population is enriched in cancer stem cells. **A** (Up) Histograms depicting the colony formation efficiency (CFE) of RMS^PR^ and RMS^CRR^ cells treated with increasing doses of ionizing radiation. Graph represents the mean of three independent experiments ± SD, one-way ANOVA. *P* values are reported in the histograms. (down) Representative images of RMS^PR^ and RMS^CRR^ cells treated with increasing doses of ionizing radiation, colonies stained with crystal violet 12 days post seeding. **B** (Up) Histograms depicting the tumorsphere forming efficiency (TFE) of RMS^PR^ and RMS^CRR^ cells treated as in (**A**). *n* = 3 independent experiments, data presented as mean values ± SD, one-way ANOVA. (Down) Representative light microscopy pictures of cancer stem cell formation assay on RMS^PR^ and RMS^CRR^ cells. *P* values are reported in the Figure: *p ≤ 0.05; **p ≤ 0.01; ***p ≤ 0.005; ****p ≤ 0.001.

As previously done for RD (FN-RMS) and RH30 (FP-RMS) cell lines [18], we generated two additional CRR cell lines (RMS^CRR^) of both FN-RMS (JR1, SMS-CTR) and FP-RMS (RH4, SCMC) by irradiating them with an ultra-hypo-fractionated schedule used for RMS patients (36 Gy total, 6 Gy/fraction) [34]. As for RD^CRR^ and RH30^CRR^ cells [18], colony-forming efficiency (CFE) assay, widely used to quantify the RT-induced cytotoxicity, was performed by irradiating adherent cells with increasing dose of RT (2-4-6 Gy). Parental RMS (RMS^PR^) cells, which were subjected to irradiation for the first time, showed a dose-dependent inhibition of the ability of single cells to form colonies, with the higher dose (6 Gy) strongly reducing colony formation (Fig. 1A). Notably, non-irradiated JR1^CRR^ and SCMC^CRR^ cells, and partly SMS-CTR^CRR^ cells, even not reaching statistical significance, more efficiently formed colonies than RMS^PR^ counterparts (Fig. 1A). Moreover, all the RMS^CRR^ cell lines, which have survived the heavy irradiation schedule, still form colonies after irradiation at different doses, with JR1^CRR^, SMS-CTR^CRR^ and SCMC^CRR^ giving rise to colonies more efficiently at least at one dose than their irradiated-RMS^PR^ counterpart (Fig. 1A). Further, JR1^CRR^ and SMS-CTR^CRR^ less efficiently proliferated than FN-RMS^PR^ cells, while no difference was found in FP-RMS (Fig. S1A). Interestingly, while all the RMS^CRR^ more efficiently repair the wound, in a wound healing assay, than their RMS^PR^ counterparts, only JR1^CRR^ reached statistical significance compared to JR1^PR^ (Fig. S1B). Since acquired radioresistance mainly depends on changes in cancer stem cell (CSC) content, the subpopulation virtually totally resistant to RT [35], we quantified floating rhabdo-spheres, tumorspheres enriched in CSCs [26] after 6 Gy of RT. All the RMS^CRR^ lines showed increased tumor-sphere forming efficiency (TFE) *versus* RMS^PR^, both in basal and irradiated conditions (Fig. 1B).

**Fig. 2:**
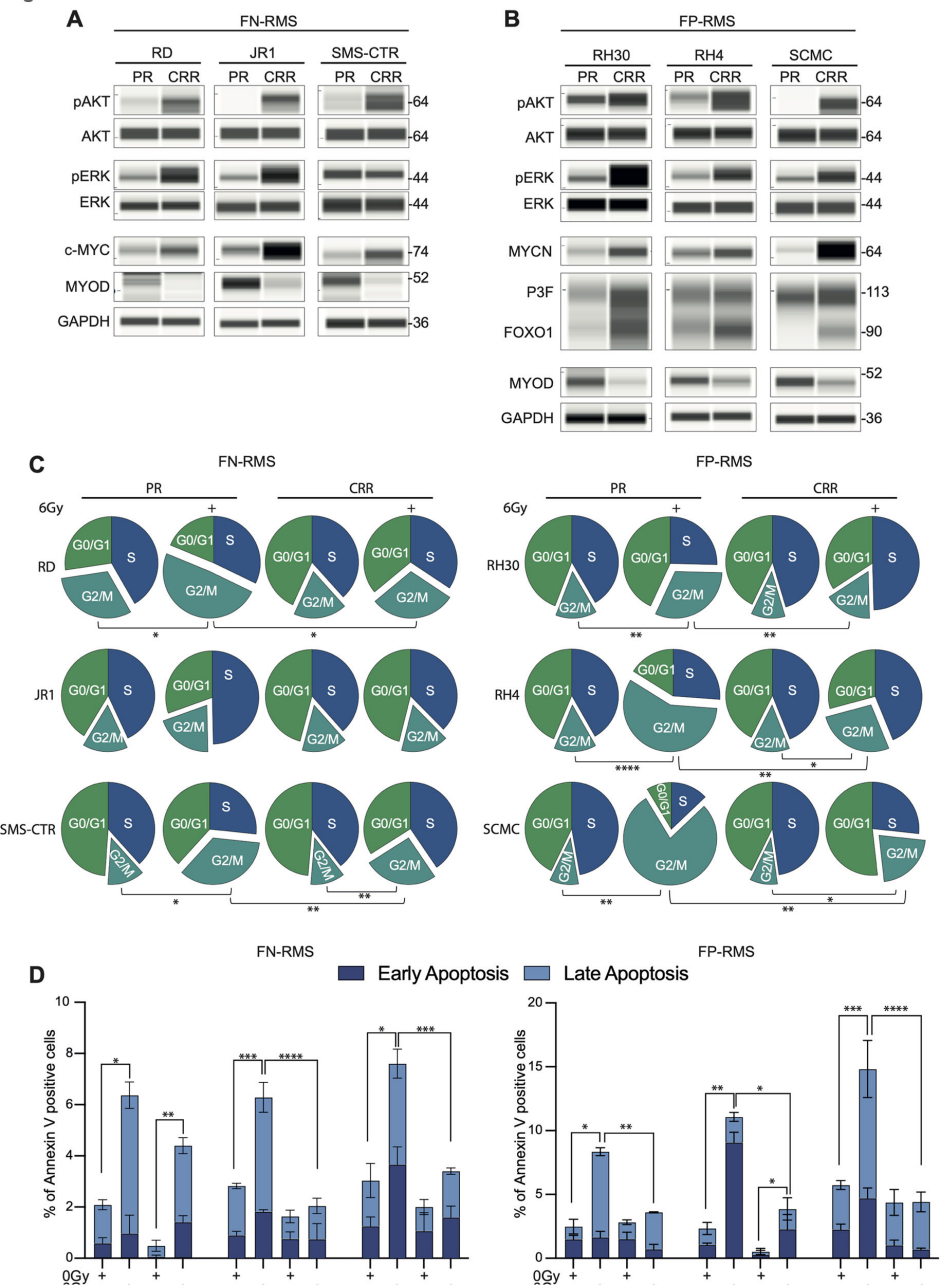
RMS^CRR^ cells are characterized by hyperactivation of pro-tumorigenic factors and escape more efficiently from IR-induced G2/M cell cycle arrest and apoptosis. **A**, **B** Representative capillary western blot (*n* = 3) depicting levels of reported proteins among RMS^PR^ and RMS^CRR^ cells. GAPDH was used as loading control. **C** Pie charts depicting the percentage of RMS^PR^ and RMS^CRR^ cells in G0/G1, S, and G2/M phases of cell cycle before and after 24 h post RT (6 Gy). The graph represents the mean of three independent experiments ± SD, one-way ANOVA. *P* values are reported on the graphs. **D** Histogram depicting the percentage of Annexin-V positive/7-AAD single- and double-positive RMS^PR^ and RMS^CRR^ cells. Graphs represent the mean of three independent experiments ± SD, one-way ANOVA. *P* values are reported in the Figure: *p ≤ 0.05; **p ≤ 0.01; ***p ≤ 0.005; ****p ≤ 0.001.

Compared to RMS^PR^, activation of survival pathways, testified by ERK and AKT phosphorylation, was higher in all RMS^CRR^, except for the phosphorylated ERKs in SMS-CTR (Fig. 2A, B) [36, 37]. Moreover, the expression levels of key genes in RMS tumorigenesis previously related to radioresistant phenotypes, such as c-MYC in FN-RMS [38], MYCN [39], and P3F [40] in FP-RMS were higher in RMS^CRR^ than RMS^PR^ cell lines (Fig. 2A, B). Conversely and in line with the enhanced stemness features, the expression levels of MYOD, the master transcription factor responsible for muscle lineage identity [41], were lower in RMS^CRR^ compared to RMS^PR^ (Fig. 2A, B). Overall, these results indicate that RMS tumor cells surviving to ultra-hypo-fractionated RT are enriched of a CSCs de-differentiated population and of activated survival pathways compared to their parental counterparts, in agreement with what has already been described [42–47].

### RMS^CRR^ cells escape more efficiently from RT-induced G2/M cell cycle arrest and apoptosis and upregulates pro-oncogenic radioresistance-related molecular signals

Since G2/M is the most radiosensitive cell cycle phase, whereas the ability to escape G2/M arrest represents a major determinant of radioresistance [48], cell cycle distribution was examined in RMS^CRR^ *versus* RMS^PR^ cells. As shown in Fig. 2C, 6 Gy of RT induced cell cycle arrest in the G2/M phase less efficiently in 5 of 6 RMS^CRR^ cell lines than in the RMS^PR^ counterpart after 24 h (Fig. 2C). Interestingly, no difference was detected between JR1^CRR^ and JR1^PR^, where RT, per se, did not significantly influence cell cycle distribution (Fig. 2C). This phenomenon was associated with resistance to RT-induced apoptosis in RMS^CRR^, as testified by the lower or unchanged percentage of apoptotic Annexin V-positive cells compared to RMS^PR^ cells in which RT markedly induced the apoptotic mark (Fig. 2D). Even in RD^CRR^ cells, which significantly enhanced the fraction of apoptotic cells after RT compared to unirradiated one, the level of increase remained under that in the irradiated FN-RMS^PR^ counterpart (Fig. 2D).

**Fig. 3:**
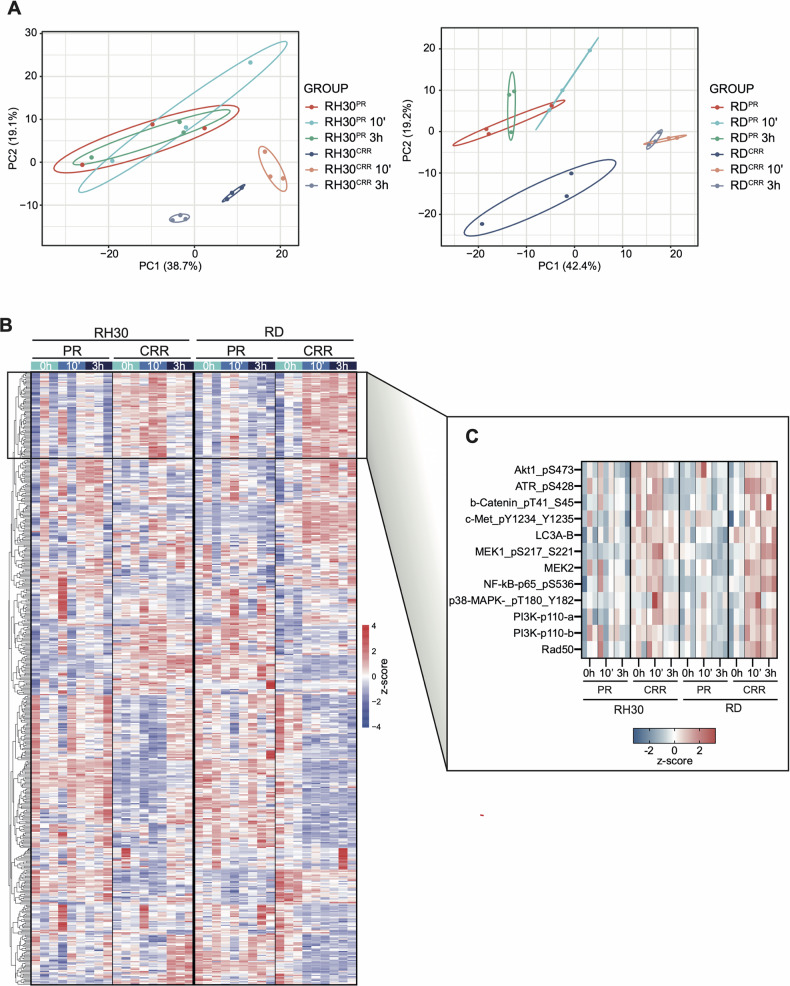
RMS^CRR^ upregulates pro-oncogenic radioresistance-related molecular signals. **A** Principal component analysis (PCA) of RPPA data of RMS^PR^ and RMS^CRR^ cells treated or not with 6 Gy of ionizing radiation and harvested at 10 min and 3 h after irradiation. **B** Cluster Heatmap depicting protein and phospho-protein modulation of RMS^PR^ and RMS^CRR^ cells treated as in (**A**). Data are reported as z-score. **C** Heatmap depicting protein and phospho-protein upregulated in RMS^PR^ and RMS^CRR^ cells treated as in (**A**).

Since the early response to RT is based on protein modifications among which phosphorylation, we evaluated protein content and phosphorylation by performing reverse phase protein array (RPPA) on RD^PR/CRR^ and RH30^PR^/^CRR^ cells treated with 6 Gy of RT and harvested at 10 min and 3 h after irradiation. Principal component analysis (PCA) revealed that RMS^CRR^ cell lines are characterized by both basal differences and different responses to irradiation *versus* RMS^PR^ (Fig. 3A). Indeed, an expression-based cluster analysis showed that both RD^CRR^ and RH30^CRR^ cells are characterized by a set of proteins or phosphoproteins whose levels were basically upregulated or induced by irradiation while appeared not modulated in RMS^PR^ ones (Fig. 3B). Interestingly, within this cluster we identified several proteins (such as LC3A-B, MEK2 and RAD50) or phosphoproteins (such as AKT1, ATR, β-Catenin, c-Met, MEK1, NF-kB, p38 and PI3K) as components of survival, proliferation and DNA repair pathways, already known to be involved in radioresistance [26, 49–55] (Fig. 3C). Altogether, this evidence suggests that acquired radioresistance improves the ability of RMS cells to escape RT-induced G2/M growth arrest and cell death and to activate pro-radioresistance molecular pathways.

### RMS^CRR^ cell lines more efficiently activate DNA Damage Response after RT

**Fig. 4:**
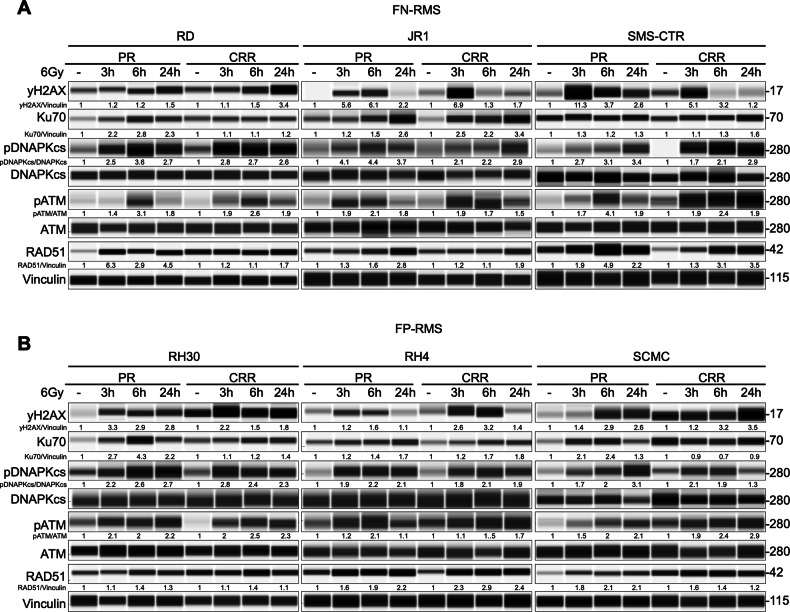
RMS^CRR^ cell lines more efficiently activate DNA damage response after RT. **A** Representative capillary western blot (*n* = 3) depicting levels of proteins involved in DNA repair among FN-RMS^PR^ and FN-RMS^CRR^ cells treated or not with 6 Gy of irradiation and harvested at 3, 6, and 24 h after irradiation. Vinculin was used as loading control. **B** Representative capillary western blot (*n* = 3) depicting levels of proteins involved in DNA repair among FP-RMS^PR^ and FP-RMS^CRR^ cells treated or not with 6 Gy of irradiation and harvested at 3, 6 and 24 h after irradiation. Vinculin was used as a loading control.

Tumor radioresistance mainly depends on the ability of cancer cells to bind and signal DSBs, through the recruitment at DSBSs sites of phosphorylated H2AX (γH2AX), a variant of the histone H2A, and to activate DNA repair, through non-homologous end joining (NHEJ) and homologous recombination (HR) [56]. The activation status of DDR was determined in cell lysates from RMS^PR^ and RMS^CRR^ after 0, 3, 6, and 24 h from RT (6 Gy). RT increased γH2AX similarly in RMS^PR^ and RMS^CRR^, persistently (3–24 h) in RD, RH30, and SCMC, transiently in JR1 (3–6 h in JR1^PR^, 3 hours in JR1^CRR^) and RH4 (3–6 h in RH4^PR^ and RH4^CRR^), persistently in SMS-CTR^PR^ (3–24 h) or transiently in SMS-CTR^CRR^ (3 h), respectively (Fig. 4A, B). RT upregulated in all cell lines the phosphorylation/activation of DNA-dependent protein kinase catalytic subunit (DNAPKcs), critical in the repair of DSBs by NHEJ [43], persistently in RD, SMS-CTR, RH30, RH4, and SCMC (3–24 h), both RMS^PR^ and RMS^CRR^ cells, whilst transiently in JR1^PR^ (3–6 h) and persistently in JR1^CRR^ (3–24 h) (Fig. 4A, B). The expression of Ku70, a protein known for recruiting DNA-PKcs to DSBs [43], was differently modulated. RT persistently upregulated Ku70 (3-6-24 h) in JR1, RH30 and RH4 across both RMS^PR^ and RMS^CRR^ (Fig. 4A, B). The upregulation was persistent in RD^PR^, transient in SCMC^PR^, while no changes were detected in RD^CRR^, SCMC^CRR^, and SMS-CTR, both RMS^PR^ and RMS^CRR^ (Fig. 4A, B). RT also upregulated the phosphorylation of ATM, a critical component of DSBs repair by HR [49], in all the cell lines, across both RMS^PR^ and RMS^CRR^ (Fig. 4A, B). Similarly, RT upregulated the expression of RAD51, a downstream effector of ATM [49], in JR1, SMS-CTR, and RH4, both RMS^PR^ and RMS^CRR^ (Fig. 4A, B). Differently, RT-induced RAD51 upregulation occurred in the RD^PR^ and RH30^PR^ but not in the RMS^CRR^ counterpart, and in SCMC^CRR^ but not in SCMC^PR^ (Fig. 4A, B).

These data suggest that RMS cells surviving high doses of RT exhibit significant, but different, alterations in their ability to signal and intervene in DNA repair. This confirms the crucial importance of the DDR molecular mechanisms for cell survival after irradiation, which preserves cellular functionality even after potentially catastrophic insults such as high doses of radiation exposure.

### RMS^CRR^ cells surviving ultra-hypofractionated RT are characterized by PRC2 hyperactivation

**Fig. 5:**
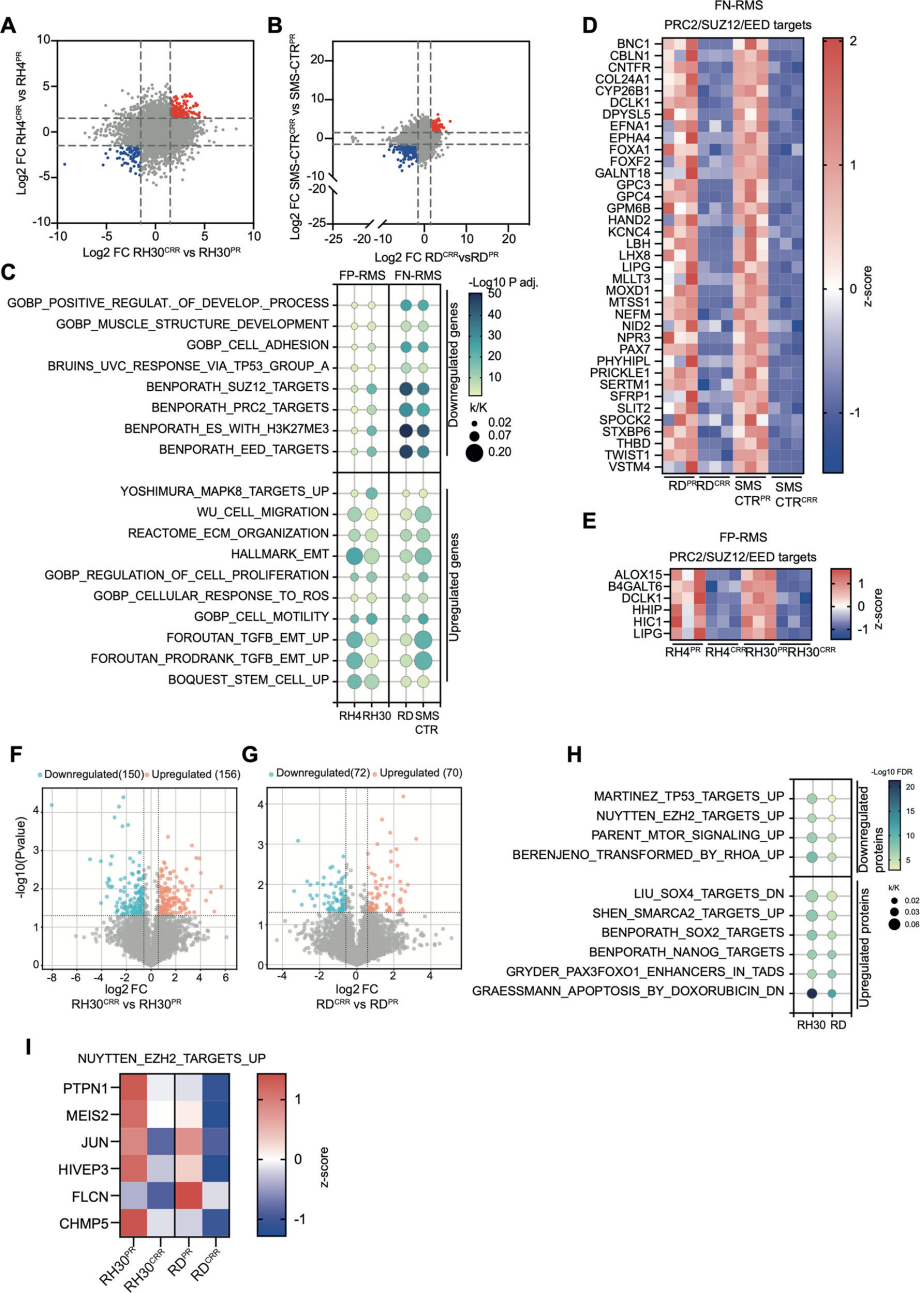
RMS^CRR^ cells rely on PRC2 activity to survive ultra-hypofractionated RT. **A**, **B** Dot blots depicting gene expression log2 Fold Change (FC) between RMS^CRR^ and RMS^PR^ cells. Red dots depict upregulated genes while the blue dots depict down-regulated genes. Dashed lines indicate log2 FC thresholds, set at +1 and −1. **C** Bubble plot depicting Gene Set Enrichment Analysis (GSEA) performed on up- and down-regulated genes in 2 FP-RMS^CRR^ cells and 2 FN-RMS^CRR^ cells compared to the PR counterparts. The size of the bubble is proportional to the enrichment significance (−Log10 P.adjusted), and the color of the bubble corresponds to k/K ratio (ratio between modulated genes and total number of genes of that geneset). **D**, **E** Heatmaps depicting gene expression levels of PRC2/SUZ12/EED targets in FN-RMS and FP-RMS cells. **F**, **G** Volcano plots depicting up- and down-regulated proteins in RH30^CRR^ and RD^CRR^ cells compared to the normal counterpart. **H** Bubble plot depicting GSEA performed on up- and down-regulated proteins in RH30^CRR^ and RD^CRR^ cells. The size of the bubble is proportional to the enrichment significance (−Log10 FDR), and the color of the bubble corresponds to k/K ratio (ratio between modulated genes and total number of genes of that geneset). **I** Heatmap depicting protein levels of common modulated EZH2 targets in RH30^CRR^ and RD^CRR^ cells.

To further characterize RMS^CRR^ cells, we analyzed the transcriptomic profiles of two FN-RMS (RD and SMS-CTR) and two FP-RMS (RH30 and RH4) cell lines to identify transcriptional changes associated with acquired radioresistance. RNA-seq analysis revealed that both FN- and FP-RMS^CRR^ cells exhibited changes in gene expression over RMS^PR^ cells, which were strongly correlated between the two cell lines of each subtype (Fig. 5A, B). Specifically, we identified 190 upregulated and 80 downregulated genes common to the two FP-RMS^CRR^ cell lines (Fig. 5A), and 130 upregulated and 213 downregulated genes common to the two FN-RMS^CRR^ cell lines (Fig. 5B). Gene Set Enrichment Analysis (GSEA) indicated significant enrichment in several pathways common to all four RMS^CRR^ cell lines linked to radioresistance. Notably, among the upregulated genes, we observed enrichment in the TGF-β signaling and epithelial-mesenchymal transition (EMT) pathways (Fig. 5C), both of which are known to play critical roles in response to RT [57]. Other enriched radioresistance-associated upregulated pathways included cellular response to reactive oxygen species (ROS) and the JNK1 (MAPK8) signaling (Fig. 5C) [58, 59]. Moreover, in line with the ability to form rhabdospheres of RMS^CRR^ compared to RMS^PR^ we reported in Fig. 1B, we noticed upregulation of a family gene associated to stemness (Fig. 5C). Interestingly, among the downregulated pathways, we found enrichment in targets of the SUZ12, EED, and PRC2 complex, as well as genes marked by the H3K27me3 histone modification on their promoter (BENPORATH_ES_WITH_H3K27ME3), suggesting increased activity of the PRC2 complex in RMS^CRR^ cell lines (Fig. 5C). Indeed, PRC2 is a chromatin remodeling complex that, through its primary enzymatic subunit EZH2, catalyzes the methylation of histone H3 at lysine 27, leading to gene repression [60]. Interestingly, distinct downregulated sets of genes known to be targeted by the PRC2 complex characterized FN- and FP-RMS^CRR^ cells (Fig. 5D, E), indicating a subtype-specific effect. Then, to assess changes in the proteomic profile of radioresistant cells, we performed mass spectrometry to compare RH30^CRR^ and RD^CRR^ cells to their parental counterparts. We identified 156 upregulated and 150 downregulated proteins in RH30^CRR^ (Fig. 5F) and 70 upregulated and 72 downregulated proteins in RD^CRR^ cells (Fig. 5G). Consistent with the transcriptomic data, GSEA revealed that genes encoding for the downregulated proteins were enriched in EZH2 targets, confirming that RMS^CRR^ cells exhibit elevated PRC2 activity at the proteomic level (Fig. 5H). Notably, among the EZH2 target downregulated proteins, only 6 were in common between RH30^CRR^ and RD^CRR^ cells (Fig. 5I), further suggesting that EZH2 could mediate cell survival after RT by regulating different targets in FN- and FP-RMS cells. Moreover, among the upregulated proteins we identified enrichment in targets of stem cell-associated genes SOX2, SOX4, and NANOG, reinforcing the idea of increased stemness properties in RMS^CRR^ cells compared to their RMS^PR^ counterparts (Fig. 5H). Taken together, these data suggest that RMS^CRR^ cells have a distinct transcriptomic and proteomic profile compared to RMS^PR^ cells, are characterized by PRC2 hyperactivity and acquire stemness molecular features.

### EZH2 promotes radioresistance directly targeting MTSS1 expression in FN-RMS^CRR^ and HHIP in FP-RMS^CRR^ cells

**Fig. 6:**
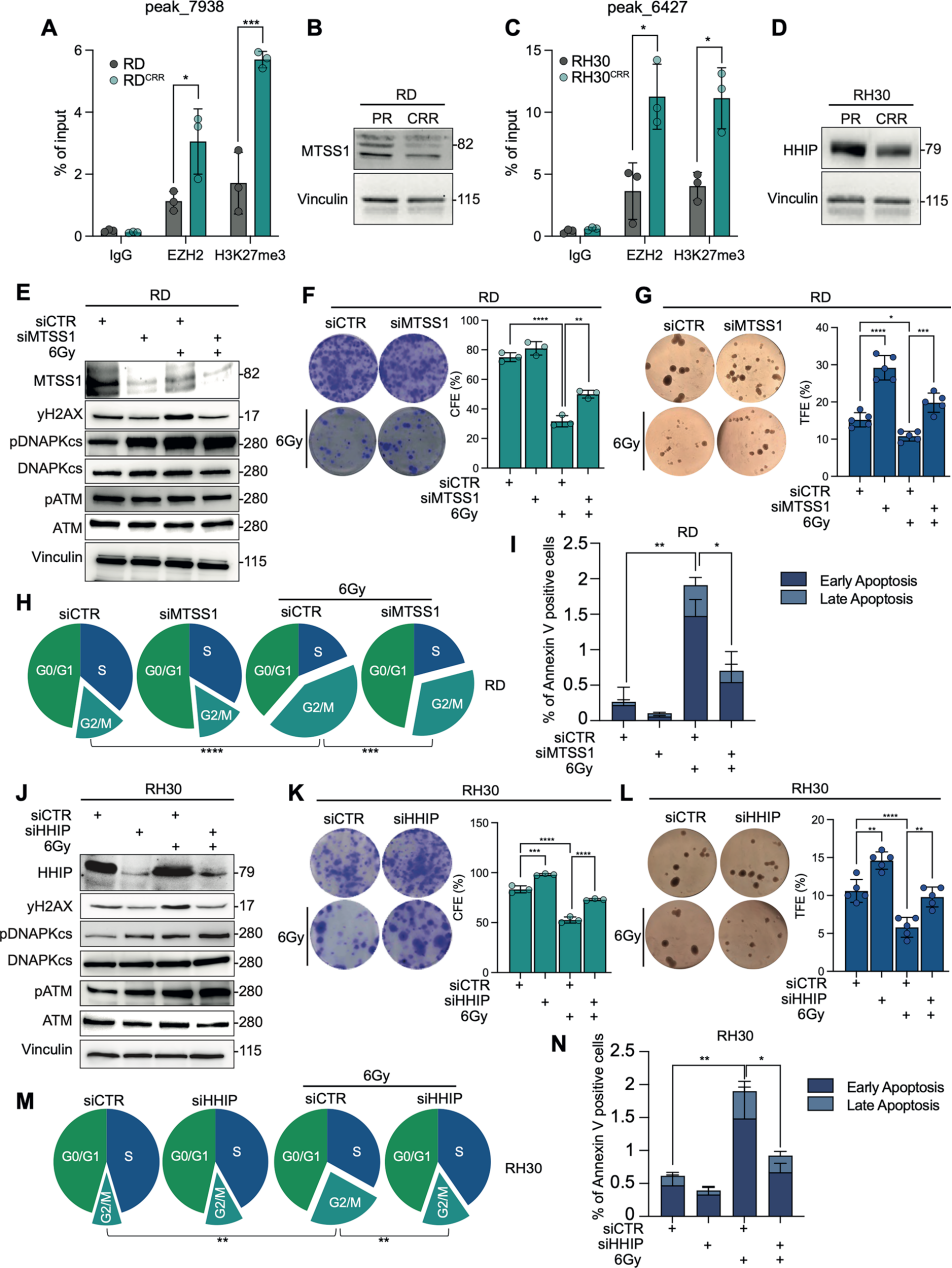
EZH2 promotes radioresistance directly targeting MTSS1 in FN-RMS^CRR^ and HHIP expression in FP-RMS^CRR^ cells. **A** Quantitative-ChIP of EZH2 and H3K27me3 in RD^PR^ and RD^CRR^ cells for peak_7938 on *MTSS1* gene. The graph represents the mean of three independent experiments ± SD, one-way ANOVA. *P* values are reported in the histograms. **B** Representative western blot (*n* = 3) depicting levels of MTSS1 in RD^PR^ and RD^CRR^. Vinculin was used as loading control. **C** Quantitative-ChIP of EZH2 and H3K27me3 in RH30^PR^ and RH30^CRR^ cells for peak_6427 on *HHIP* gene. Graph represents the mean of three independent experiments ± SD, one-way ANOVA. *P* values are reported in the histograms. **D** Representative western blot (*n* = 3) depicting levels of HHIP in RH30^PR^ and RH30^CRR^. Vinculin was used as loading control. **E** Representative western blot (*n* = 3) depicting levels of MTSS1, γH2AX, pDNAPKcs, total DNAPKcs, pATM, and total ATM in RD^PR^ silenced for MTSS1 and treated with 6 Gy of irradiation alone or in combination. Vinculin was used as loading control. **F** Left: Representative images of RD^PR^ colonies treated as in (**E**) and stained with crystal violet 12 days post-seeding. Right: Histograms depicting the CFE of RD^PR^ treated as in (E). Graph represents the mean of three independent experiments ± SD, one-way ANOVA. *P* values are reported in the histograms. **G** Left: Representative light microscopy pictures of cancer stem cell formation assay on RD^PR^ cells treated as in (**E**). Right: Histograms depicting the tumorsphere forming efficiency (TFE) of RD^PR^ cells treated as in (**E**). *n* = 3 independent experiments, data presented as mean values ± SD, one-way ANOVA. **H** Pie charts depicting the percentage of RD^PR^ cells, treated as in (**E**), in G0/G1, S, and G2/M phases of cell cycle. Graph represents the mean of three independent experiments, one-way ANOVA. *P* values are reported on the graphs. **I** Histograms depicting the percentage of Annexin-V positive/7-AAD single- and double-positive RD^PR^ cells treated as in (**E**). Graphs represent the mean of three independent experiments ± SD, one-way ANOVA. **J** Representative western blot (*n* = 3) depicting levels of HHIP, γH2AX, pDNAPKcs, total DNAPKcs, pATM and total ATM in RH30^PR^ silenced for HHIP and treated with 6 Gy of irradiation alone or in combination. Vinculin was used as loading control. **K** Left: Representative images of RH30^PR^ colonies treated as in (**E**), colonies stained with crystal violet 12 days post seeding. Right: Histograms depicting the CFE of RH30^PR^ treated as in (**J**). Graph represents the mean of three independent experiments ± SD, one-way ANOVA. *P* values are reported in the histograms. **L** Left: Representative light microscopy pictures of cancer stem cell formation assay on RH30^PR^ cells treated as in (**J**). Right: Histograms depicting the tumorsphere forming efficiency (TFE) of RH30^PR^ cells treated as in (**J**). *n* = 3 independent experiments, data presented as mean values ± SD, one-way ANOVA. **M** Pie charts depicting the percentage of RH30^PR^ cells, treated as in (**J**), in G0/G1, S, and G2/M phases of cell cycle. Graph represents the mean of three independent experiments, one-way ANOVA. *P* values are reported on the graphs. **N** Histograms depicting the percentage of Annexin-V positive/7-AAD single- and double-positive RH30^PR^ cells treated as in (**J**). Graphs represent the mean of three independent experiments ± SD, one-way ANOVA. *p ≤ 0.05; **p ≤ 0.01; ***p ≤ 0.005; ****p ≤ 0.001.

To investigate how the PRC2 complex might be involved in the mechanisms of acquired radioresistance in RMS^CRR^ cells, we first validated the downregulation of several EZH2 target genes by qRT-PCR. In agreement with the RNA-seq data, we showed that *MTSS1*, *CNTFR*, *GPC3*, *GPC4*, *PAX7* and *PRICKLE1* are downregulated in FN-RMS^CRR^ cells compared with FN-RMS^PR^ cells (Fig. S2A). On the other hand, we also confirmed the downregulation of *HHIP* and *ALOX15* in FP-RMS^CRR^ cells compared with FP-RMS^PR^ cells (Fig. S2B). To identify direct EZH2 targets, we then analyzed public ChIP-seq data for EZH2 in SMS-CTR FN-RMS cells. Interestingly, we observed EZH2 enrichment on putative regulatory regions of the *MTSS1* (Fig. S2C) and *HHIP* genes (Fig. S2D). We therefore analyzed EZH2 binding and H3K27me3 levels in our RMS^CRR^ models by quantitative ChIP, compared with their RMS^PR^ counterparts. Notably, in RD^CRR^ cells, EZH2 binding and H3K27me3 levels at the *MTSS1* locus were increased compared with RD^PR^ cells (Fig. 6A), and this was reflected in reduced MTSS1 protein levels in RD^CRR^ versus RD^PR^ cells (Fig. 6B). Similarly, we analyzed EZH2 binding and H3K27me3 levels at *HHIP* in RH30^CRR^ cells compared with RH30^PR^ cells. Also in this case, we observed increased EZH2 binding at *HHIP* and higher H3K27me3 levels (Fig. 6C), resulting in decreased HHIP protein levels in RH30^CRR^ cells (Fig. 6D). Interestingly, *MTSS1* silencing in combination with RT in RD^PR^ cells was able to counteract the RT-induced increase in γH2AX levels, leading to enhanced phosphorylation of DNAPKcs and thus increasing the efficiency of DNA damage repair (Fig. 6E). Next, we investigated whether *MTSS1* silencing could render RD^PR^ cells more radioresistant by analyzing colony-forming efficiency and stemness. Our results indicate that *MTSS1* knocking down significantly counteracts the RT-induced reduction in both colony number (Fig. 6F) and the CSC population (Fig. 6G). Notably, *MTSS1* silencing alone was sufficient to increase the stemness of RD^PR^ cells (Fig. 6G). Furthermore, the combination of *MTSS1* depletion and RT had evident effects on cell cycle progression and apoptosis. Indeed, *MTSS1* silencing was able to prevent both the G2/M cell cycle arrest (Fig. 6H) and the induction of apoptosis (Fig. 6I) significantly promoted by RT alone. *HHIP* silencing in combination with RT was able to counteract the RT-induced accumulation of γH2AX in RH30^PR^ cells, by promoting increased phosphorylation of both DNAPKcs and ATM (Fig. 6J), suggesting enhanced DNA repair efficiency. We then tested whether *HHIP* knocking down could render the cells more radioresistant by analyzing colony-forming ability and stemness. Again, we observed that *HHIP* depletion significantly counteracted the RT-dependent reduction in both colony-forming efficiency (Fig. 6K) and the CSC population (Fig. 6L). Notably, *HHIP* silencing alone was sufficient to promote an increase in both colony number and the CSC population. In addition, *HHIP* depletion was able to counteract both the accumulation of cells in G2/M (Fig. 6M) and the RT-dependent induction of apoptosis (Fig. 6N).

Taken together, these data demonstrate that EZH2 displays increased activity on *MTSS1* and *HHIP* genes in FN-RMS^CRR^ and FP-RMS^CRR^ cells, respectively, compared with their parental counterparts, resulting in reduced expression of these genes that may be responsible for the enhanced radioresistance of RMS^CRR^ cells.

### EZH2 pharmacological inhibition counteracts acquired radioresistance in vitro

**Fig. 7:**
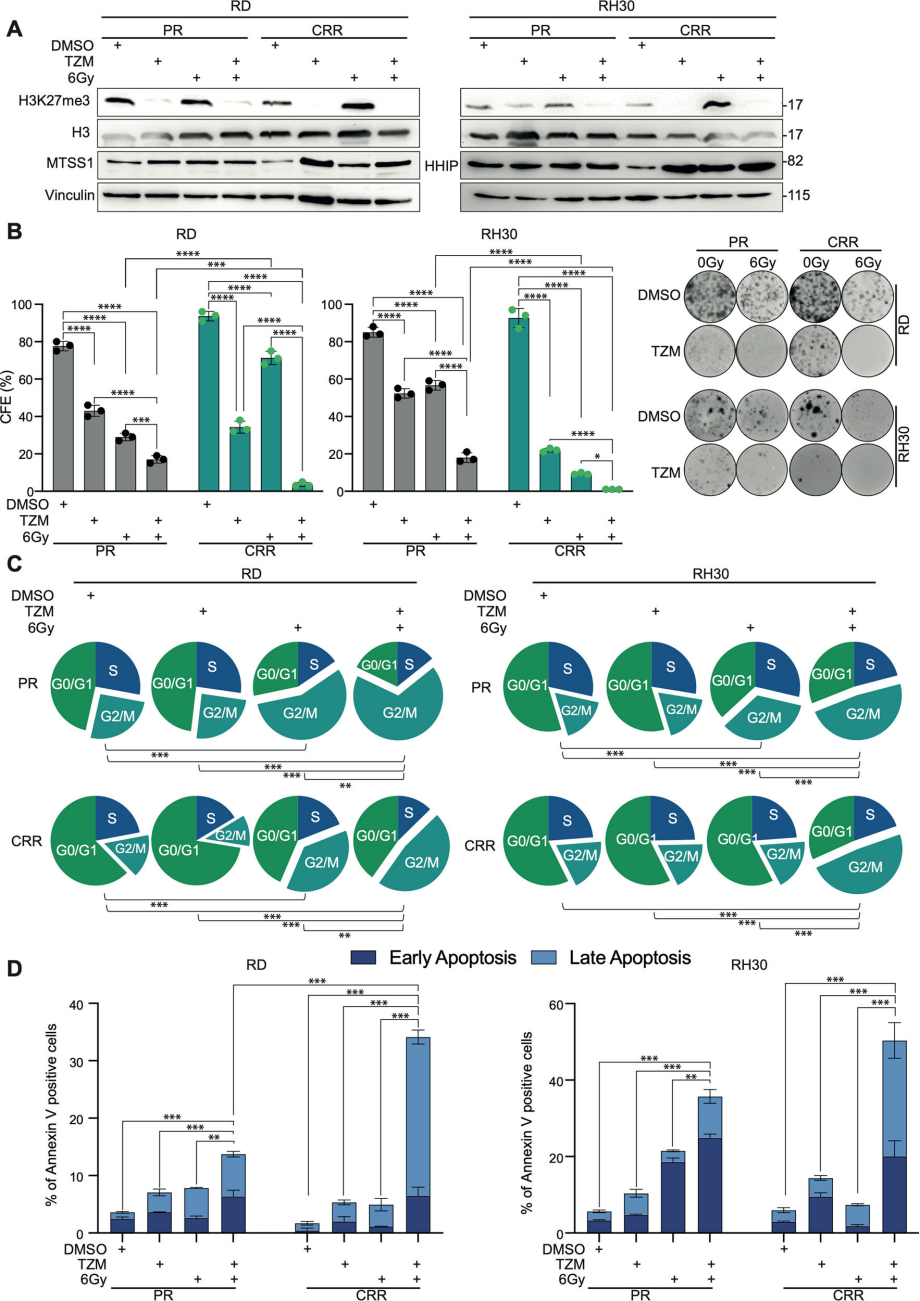
EZH2 inhibition counteracts intrinsic and acquired radioresistance in vitro. **A** Representative western blots (*n* = 3) depicting levels of H3K27me3 among RMS^PR^ and RMS^CRR^ cells treated with 6 Gy of irradiation and 10 μM of TZM alone or in combination. Total histone H3 was used as loading control. **B** (Left) Histograms depicting the CFE of RD and RH30 PR and CRR treated with 6 Gy of irradiation or 10 μM of TZM alone or in combination. Graphs represent the mean of three independent experiments ± SD, one-way ANOVA. *P* values are reported in the histograms. (Right) Representative images of RD and RH30, PR and CRR cells treated with 6 Gy of irradiation or 10 μM of TZM alone or in combination, colonies stained with crystal violet 12 days post seeding. **C** Pie charts depicting the percentage of RMS^PR^ and RMS^CRR^ cells, treated as in (**A**), in G0/G1, S, and G2/M phases of cell cycle. Graphs represent the mean of three independent experiments, one-way ANOVA. P values are reported on the graphs. **D** Histograms depicting the percentage of Annexin-V positive/7-AAD single- and double-positive RMS^PR^ and RMS^CRR^ cells treated as in (**A**). Graphs represent the mean of three independent experiments ± SD, one-way ANOVA. *P* values are reported in the Figure: *p ≤ 0.05; **p ≤ 0.01; ***p ≤ 0.005; ****p ≤ 0.001.

**Fig. 8:**
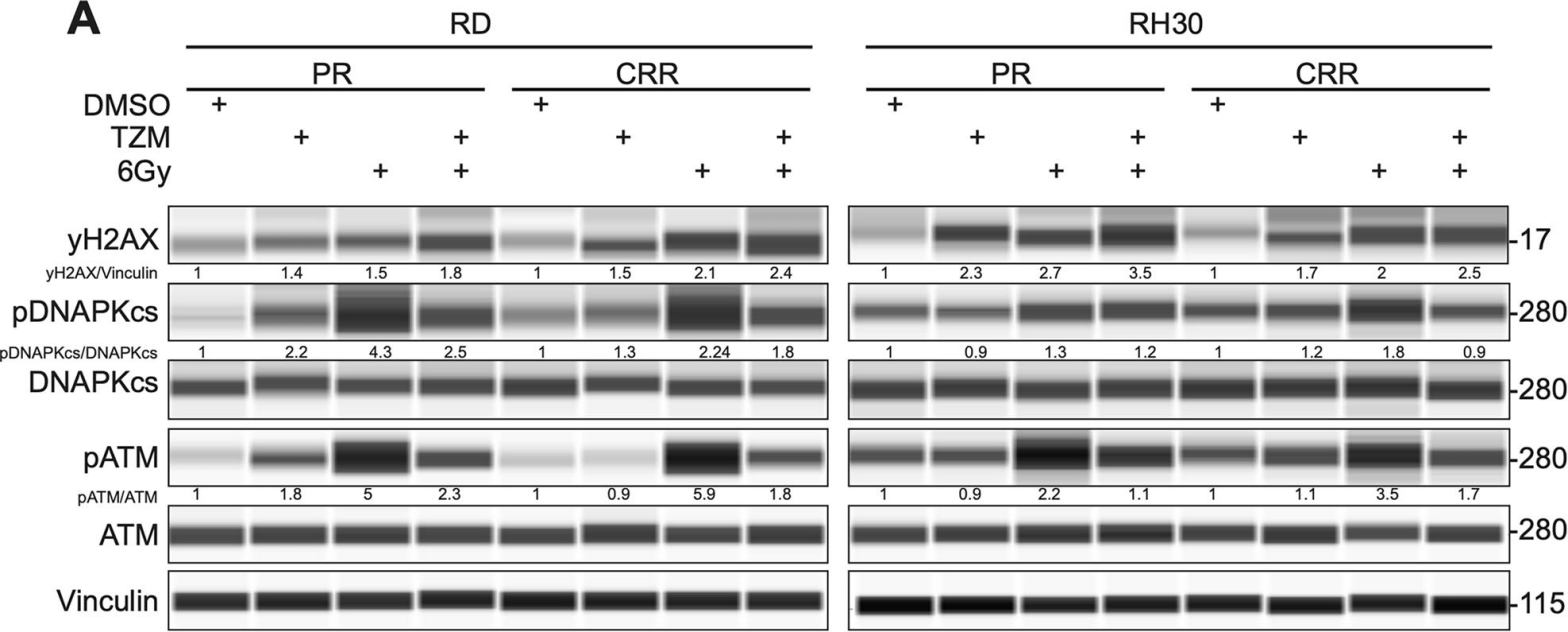
TZM counteracts the activation of DNA repair. Representative capillary western blots (*n* = 3) depicting levels of proteins involved in DNA repair among RMS^PR^ and RMS^CRR^ cells treated with 6 Gy of irradiation or 10 μM of TZM alone or in combination. Vinculin was used as loading control.

To evaluate the PRC2 complex as a valuable target to enhance the efficacy of RT and overcome radioresistance, we firstly investigated the expression of *EZH2*, *SUZ12*, and *EED* in RMS patients. Analysis of public datasets revealed that all three PRC2 components were overexpressed in RMS patients compared to the normal skeletal muscle tissues as controls (Fig. S3A), confirming data on other patients’ datasets previously reported [61, 62]. In addition, *SUZ12* low expression was significantly associated with a good prognosis in both RMS subtypes, while that of *EZH2* and *EED* significantly correlated with a good prognosis only in FP-RMS patients (Fig. S3B), which is the most radioresistant subtype. Interestingly, the effects of CRISPR/Cas9 knockout (KO) of *EZH2*, *SUZ12* and *EED* on the survival of RMS cell lines in DepMap portal (Achilles project, https://depmap.org/portal/achilles) showed that RMS cells are only mildly dependent on PRC2 components’ expression (Fig. S3C), suggesting that their targeting might be more effective in combinatorial therapies than as a standalone approach. We then decided to investigate the effects of Tazemetostat (TZM), an investigational first-in-class targeted epigenetic regulator that specifically inhibits EZH2 activity, in PR and CRR RD and RH30 cells [63]. Treatment with TZM resulted in significantly higher inhibition of cell viability in RD^CRR^ and RH30^CRR^ cells compared to RH30^PR^ at 72 h, as demonstrated by the significantly lower drug dose needed to reduce cell viability by 50% (IC_50_) (Fig. S4A, B). This suggested a greater dependency on EZH2 activity for radioresistant cells. We confirmed this data also using another EZH2 inhibitor, 3-Deazaneplanocin A (DZNeP) (Fig. S4C, D). After having validated TZM efficiency in preventing EZH2 activity at 10 μM, i.e., the dose around that of the RMS^CRR^ IC_50_, detecting H3K27me3 levels downregulation in all the tested cell lines (Fig. 7A), we assessed the ability of the drug to radiosensitize RMS cells. To this end, we demonstrated that, blocking EZH2 activity by TZM treatment led to increase MTSS1 and HHIP protein levels in RD^CRR^ and RH30^CRR^ cells, respectively (Fig. 7A). Moreover, colony formation assay was performed on tumor cells pretreated for 24 h with the drug (10 μM) and then irradiated with a dose of 6 Gy. As shown in Fig. 7B, TZM as a single agent reduced the CFE of FN-RMS and FP-RMS cells, both PR and CRR. Moreover, TZM markedly radiosensitized both the parental and the radioresistant cells, with the latter responding more efficiently (Fig. 7B). Further, TZM alone did not significantly change the cell cycle distribution, whilst, when combined with RT, it induced a significant cell accumulation in the G2/M phase compared to single treatments in both RMS^PR^ and RMS^CRR^ cells (Fig. 7C). Similarly, TZM as a single agent did not significantly affect the percentage of the apoptotic cells, whereas pre-treatment with the drug in combination with RT markedly increased apoptosis in both RMS^PR^ and RMS^CRR^ (Fig. 7D). Again, RMS^CRR^ showed the higher apoptotic marker positivity (Fig. 7D). While the basal levels of γH2AX, crucial DNA damage marker, was expressed similarly, it was increased by both TZM and RT as single treatments, with the former more effective, and further augmented by the combination 6 hours after RT in all the cell lines, except that in RH30^CRR^. Evaluating the DNA repair pathway activation, responsible for the survival of DNA-damaged cells, we found that the increased phosphorylation/activation of DNAPKcs, a key player in NHEJ, induced by RT was counteracted by TZM in both RD^PR^ and RD^CRR^, and in RH30^CRR^, whilst no effects were described in RH30^PR^ (Fig. 8). Similarly, TZM counteracted RT-induced phosphorylation/activation of ATM, which is crucial for HR, in both RMS^PR^ *versus* RMS^CRR^ cells (Fig. 8).

Collectively, these findings demonstrate that TZM radiosensitizes both FN- and FP-RMS parental cells by promoting DNA damage and counteracting DSB repair, affecting their acquired radioresistance in vitro.

### TZM in vivo radiosensitizes RMS^PR^ xenografts and counteracts tumor growth of RMS^CRR^

**Fig. 9:**
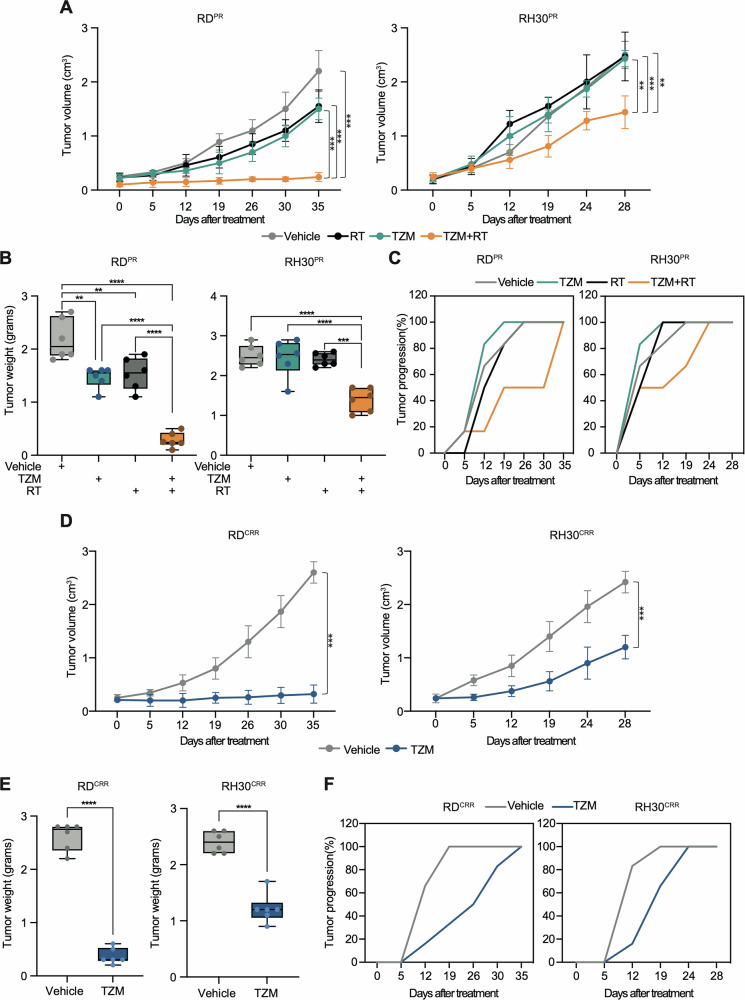
TZM in vivo sensitizes RMS^PR^ cells to radiotherapy and counteracts RMS^CRR^ growth*.* **A** Tumor volume of RD^PR^ and RH30^PR^ xenografts pre-treated with 75 mg/Kg of TZM assessed by caliper measurement represented in mm^3^. Xenografted mice were pre-treated for 5 days with 75 mg/Kg of TZM and then irradiated each 2 days for 3 times with a single dose of 2 Gy of RT. Data presented as mean values ± SD, two-way ANOVA. **B** Tumor weight of transplanted RD^PR^ and RH30^PR^ cell xenografts treated as in (**A**). Data presented as mean values ± SD, two-way ANOVA. **C** Kaplan–Meier estimates for rates of progression for mice treated as in (**A**). **D** Tumor volume of RD^CRR^ and RH30^CRR^ xenografts treated with 75 mg/Kg of TZM assessed by caliper, measurement represented in mm^3^. Data presented as mean values ± SD, two-way ANOVA. **E** Tumor weight of transplanted RD^CRR^ and RH30^CRR^ cell xenografts treated as in (**A**). Data presented as mean values ± SD, two-way ANOVA. **F** Kaplan–Meier estimates for rates of progression for mice treated as in (**A**). P values are reported in the Figure: **p ≤ 0.01; ***p ≤ 0.005; ****p ≤ 0.001.

To assess the ability of TZM to hinder in vivo the intrinsic radioresistance of RMS, RD^PR^ and RH30^PR^ cells were subcutaneously injected in nude mice. TZM (75 mg/kg [64] or vehicle were administered intraperitoneally (i.p.) once daily for 5 consecutive days before irradiation. Mice belonging to Vehicle+RT and TZM + RT groups were irradiated with the dose of 2 Gy the 1st, 3rd, and 5th day [65, 66], one hour after receiving TZM, for a total dose of 6 Gy. Tumor volumes were measured at the time points shown in Fig. 9A. While both RT or TZM single treatments mildly reduced tumor growth of RD cells and were ineffective in RH30 cells, the combination of TZM and RT completely prevented RD xenografts growth and reduced that of RH30 ones even if they continued to grow progressively throughout the experiment (Figs. 9A and S5A). Tumor weights were markedly reduced in mice receiving the combined TZM and RT treatment compared to untreated controls or those given either therapy alone (Fig. 9B). Moreover, analysis of tumor progression, defined as a doubling in tumor volume, demonstrated that the TZM and RT co-treatment significantly delayed tumor progression more effectively than individual treatments (Fig. 9C).

To verify whether radioresistant cells had acquired vulnerability to EZH2 inhibition in vivo we tested TZM (75 mg/kg) as single treatment on RMS^CRR^ xenografts. As shown in Figs. 9D and S5B, compared to untreated mice, TZM completely hampered tumor growth of RD^CRR^ xenografts and significantly reduced that of RH30^CRR^ ones. Accordingly, tumor weights of xenografts from mice treated with TZM decreased significantly compared to those of untreated mice (Fig. 9E), as well as tumor progression (Fig. 9F). Altogether, these findings underscore the efficacy of TZM to enhance the radiosensitivity of RMS cells and to counteract that of RMS cells that survive hypo-fractionated RT.

## Discussion

Through an integrated multi-omics approach, including transcriptomics, proteomics, and phosphoproteomics, we systematically profiled the molecular reprogramming underlying acquired radioresistance in both FN- and FP-RMS cells. This strategy enabled the identification of subtype-specific and shared adaptations involving transcriptional reprogramming, altered signaling networks, and changes in DNA damage response, stemness, and cell cycle control. Importantly, this integrative analysis uncovered pharmacologically actionable vulnerabilities, including enhanced dependency on the histone methyltransferase EZH2.

Transcriptomic analyses of RMS^CRR^ *versus* RMS^PR^ [18] revealed the activation of pathways commonly associated with radioresistance, such as TGF-β signaling and EMT. Both are implicated in driving CSC traits, invasiveness, and therapeutic evasion that are critically involved in RMS persistence, metastasis, and resistance to therapies, including RT [26]. Thus, they could represent a cell population that selectively survive RT in RMS^CRR^ giving raise to the acquired redioresitance phenotype. Increased activity of oxidative stress response pathways, specifically involving ROS and JNK1, was also observed, consistent with the role of JNK signaling as a downstream effector of TGF-β in irradiation contexts [67, 68]. Notably, among the downregulated gene families in CRR cells, we observed a significant enrichment of targets of the components of PRC2 complex (EZH2, SUZ12, EED) and genes marked by H3K27me3, suggesting PRC2 hyperactivation in acquired radioresistance. EZH2 oncogenic role in RMS has been well established by previous studies [23, 69–71]. Intriguingly, a downregulation of distinct sets of PRC2-targeted genes was highlighted in FN- and FP-RMS^CRR^ cells suggesting subtype-specific gene modulation, confirming the molecular differences between the two RMS subtypes [71]. EZH2 has been recently related to radioresistance and, in line, its inhibition has been reported to increase radiosensitivity [23] and reduce resistance to therapy [72]. In particular, it has recently been shown that EZH2 inhibition can reverse acquired radioresistance in models of bladder cancer [23] and head and neck cancer cells by impairing DNA repair, promoting autophagy, and increasing sensitivity to glucose starvation [73]. Additionally, it has been also reported that EZH2 expression positevely correlates with post-irradiation recurrence in prostate cancer [74] and breast cancer patients [25].

Proteomic and phosphoproteomic profiling via RPPA further uncovered distinct signaling rewiring in RMS^CRR^ cells. Increased activation of ATR, AKT, and the MEK/ERK/p38 axis was observed both basally and in response to radiation. ATR phosphorylation in G1, typically less responsive to RT-induced DNA damage, suggests an adaptation aimed at promoting radioresistance [75]. The enhanced activity of the MEK/ERK/p38 axis is known to stabilize MYC proteins, which we found upregulated in RMS^CRR^ cells independently of RT. While in RMS^PR^ cells, MYC/N-MYC levels increased only upon irradiation, in RMS^CRR^ cells, they were already elevated at baseline and remained stable post-RT. This shift toward constitutive MYC activation may promote CSC maintenance and resistance to apoptosis [76]. Moreover, activation of AKT was accompanied by phosphorylation of NF-κB and β-catenin, suggesting convergence of survival and stemness-promoting pathways in RMS^CRR^ cells [77]. This signaling landscape is consistent with the phenotypic enrichment of CSC-like populations in both FN- and FP-RMS^CRR^ cells, as shown by increased tumorsphere formation and expression of stemness-related markers. CSCs are well-established contributors to radioresistance and disease recurrence, and their expansion may represent a key mechanism of therapy failure in RMS. Cell cycle analysis revealed that RMS^CRR^ cells fail to accumulate in G2/M following RT, instead exhibiting G1 retention. Given that G2/M is the most radiosensitive phase [43], this cell cycle redistribution may enable escape from RT-induced cytotoxicity. Furthermore, RMS^CRR^ cells showed attenuated RT-induced apoptosis, suggesting increased damage tolerance and survival. These findings underscore the multifactorial adaptations RMS cells undergo to acquire and maintain a radioresistant phenotype.

To mechanistically link EZH2 hyperactivation to radioresistance, we identified and validated *MTSS1* in FN-RMS and *HHIP* in FP-RMS as its subtype-specific target genes. Public ChIP-seq data (GEO: GSE85171) and quantitative ChIP assays confirmed increased EZH2 binding and H3K27me3 deposition at these loci in RMS^CRR^ cells, resulting in reduced protein expression. Functional silencing of *MTSS1* or *HHIP* in RMS^PR^ cells recapitulated the resistant phenotype, reducing RT-induced γH2AX accumulation, enhancing ATM/DNA-PKcs activation, restoring clonogenicity and CSC features, as well as preventing G2/M arrest and apoptosis. These findings establish a causal epigenetic axis in which EZH2-mediated repression of *MTSS1* and *HHIP* enhances DNA repair efficiency and stemness, thereby promoting both intrinsic and acquired radioresistance in RMS. Consistently, MTSS1 and HHIP are well-established tumor suppressor proteins that have been linked in promoting resistance to therapies in other tumor context [78, 79].

To exploit the PRC2 dependency uncovered by our multi-omics analysis, we tested the effects of the EZH2 inhibitor Tazemetostat (TZM), a first-in-class selective inhibitor of EZH2 currently under clinical investigation on several pediatric cancers among which sarcomas (NCT02601937, NCT04917042, and NCT03213665) [80], to evaluated its antitumor efficacy both as monotherapy and in combination with RT. Notably, RMS^CRR^ cells displayed increased sensitivity to TZM monotherapy, with reduced IC_50_ values compared to RMS^PR^ cells. To do so, we used a dose of TZM corresponding to that of FP-RMS^CRR^ cells IC_50_, which was about ½ of that in RMS^PR^ cells, also discovering that RMS^CRR^ cells were more dependent from EZH2 activity compared to the parental ones. We demonstrate that TZM treatment as monotherapy or with RT led to increased MTSS1 and HHIP protein levels in RD^CRR^ and RH30^CRR^ cells, respectively. Moreover, co-treatment with TZM and RT amplified the inhibition of tumor cells to form colonies as single cells, expanded in RMS^PR^ cells and restored in RMS^CRR^ cells the accumulation in the G2/M cell cycle phase contemporary reducing that in G1, and markedly intensified apoptosis especially in RMS^CRR^ cells. Noteworthy, TZM alone did not significantly alter the distribution of cell cycle phases or apoptosis in either RMS^PR^ or RMS^CRR^ cells. This insight aligns with previous studies showing that agents promoting G2/M arrest can synergize with RT to enhance tumor cell killing compared to RT alone, supporting their potential as radiosensitizing molecules [81]. In particular, TZM shows a stronger radiosensitizing effect on RMS^CRR^ cells compared to RMS^PR^, indicating its potential to overcome acquired radioresistance. However, the TZM dose lower than IC_50_ as a single agent did not affect RMS^PR^ cell survival but promoted radiosensitization in vitro. This observation is in accordance with the modest dependency level of RMS cells from EZH2/SUZ12/EED level obtained from DepMap.

The potential of EZH2 inhibition as a radiosensitizing strategy in RMS is supported by the evidence that TZM combined with RT significantly increased γH2AX levels in both RMS^PR^ and RMS^CRR^ cells, while reducing ATM activation in both and DNAPKcs in RMS^CRR^ compared to single treatments, as key mediators of HR and NHEJ repair pathways, respectively [43, 49]. These results indicate impaired DNA repair efficiency, possibly linked to reduced G1 phase accumulation [82, 83]. Histone methylation is known to influence radiosensitivity by affecting DNA repair [84]; EZH2-mediated H3 methylation promotes repair of radiation-induced DSBs, thus limiting DNA damage accumulation. Consistently, EZH2 inhibition by TZM reduced H3K27me3 levels and enhanced RT-induced DNA damage in RMS cells.

Finally, we provide evidence that TZM significantly enhances the therapeutic impact of RT in RMS^PR^ cells in preclinical models of parental RMS cells. Indeed, while TZM as a single agent only slightly reduced tumor growth in FN-RMS^PR^ and did not affect that of FP-RMS^PR^, the combination with RT completely inhibited tumor progression in the former and significantly reduced tumor growth in the latter. The difference of the efficacy of the combined therapy could be explained by the more radioresistant nature of the FP-RMS subtype compared to the FN-RMS. Moreover, our in vivo data also show that TZM monotherapy effectively counteracts the growth of radioresistant RMS xenografts, preventing the growth of FN-RMS^CRR^ cells and significantly decreasing that of FP-RMS^CRR^ xenografts compared to untreated controls.

Overall, our findings demonstrate that RMS cells undergo subtype-specific transcriptional and post-translational reprogramming to evade RT-induced cytotoxicity. Through a comprehensive multi-omics strategy, we identified EZH2 as a key regulator of the radioresistant phenotype and a promising therapeutic target. By impairing DDR efficiency, promoting G2/M accumulation, and reactivating apoptotic responses, EZH2 inhibition with TZM enhances the efficacy of RT and suppresses the growth of both treatment-naïve and resistant RMS cells. These results support the incorporation of EZH2 inhibitors into RMS therapeutic regimens, particularly in recurrent disease where re-irradiation may not be feasible. Future clinical studies should investigate whether EZH2 inhibition can improve outcomes for patients with high-risk or treatment-resistant RMS.

## Supplementary information

Supplementary Figures

Uncropped western blots

## Data Availability

All the datasets analyzed in this work are publicly available. Affymetrix data from RMS patients in Supplementary Fig. 2A and correlation data between patients’ survival and gene expression in Supplementary Fig. 2B were obtained from R2 Genomics Analysis and Visualization Platform (https:// hgserver1.amc.nl/cgi-bin/r2/main.cgi) using Williamson dataset (E-TABM-1202) and Asman dataset (GSE9103). Cell dependency data in Supplementary Fig. 2C was downloaded from DepMap portal (https://depmap.org/portal/). All the transcriptomic and proteomic data obtained in this work are available under request.
