# Supplementary material for: Multi-omics analyses identify EZH2 as a central driver in rhabdomyosarcoma radioresistance and highlight Tazemetostat as an effective radiosensitizer in vitro and in vivo
Source: Cell Death Dis. 2026 Jun 1;17(1):683. doi: 10.1038/s41419-026-08938-0 (PMC13439469; doi:10.1038/s41419-026-08938-0)
Supplement: Supplementary file 1 [file 41419_2026_8938_MOESM1_ESM.pdf]

Supplementary Figure 1

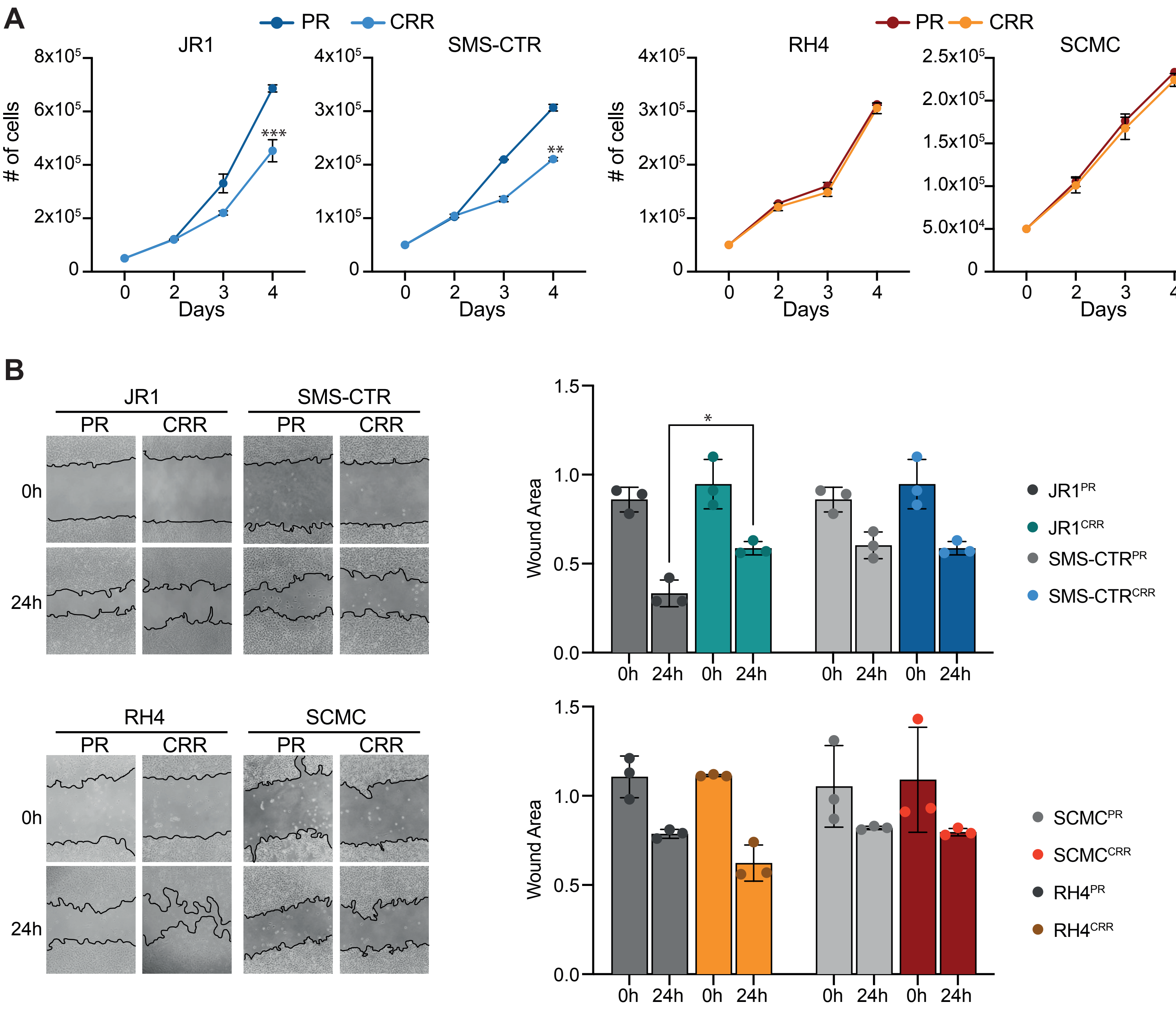

Supplementary Figure 1. RT survived RMS cell population has increased migration potential. **A)** Growth-curve analysis of RMS^{PR} and RMS^{CRR} cells. Graphs represent the mean of three independent experiments ± SD, two-tailed t-test. P values are reported in the graphs. **B)** Wound healing experiments in RMS^{PR} and RMS^{CRR} cells. A scratch was made at time 0 and maintained for 24 hours. The dotted lines represent the edges of the wound. Representative images (left) were acquired under light microscope (10x magnification). (Right) Histograms depicting the migration index reported as the % of wound area.Graph represents the mean of three independent experiments ± SD, two-tailed t-test. P values are reported in the histograms.

Supplementary Figure 2

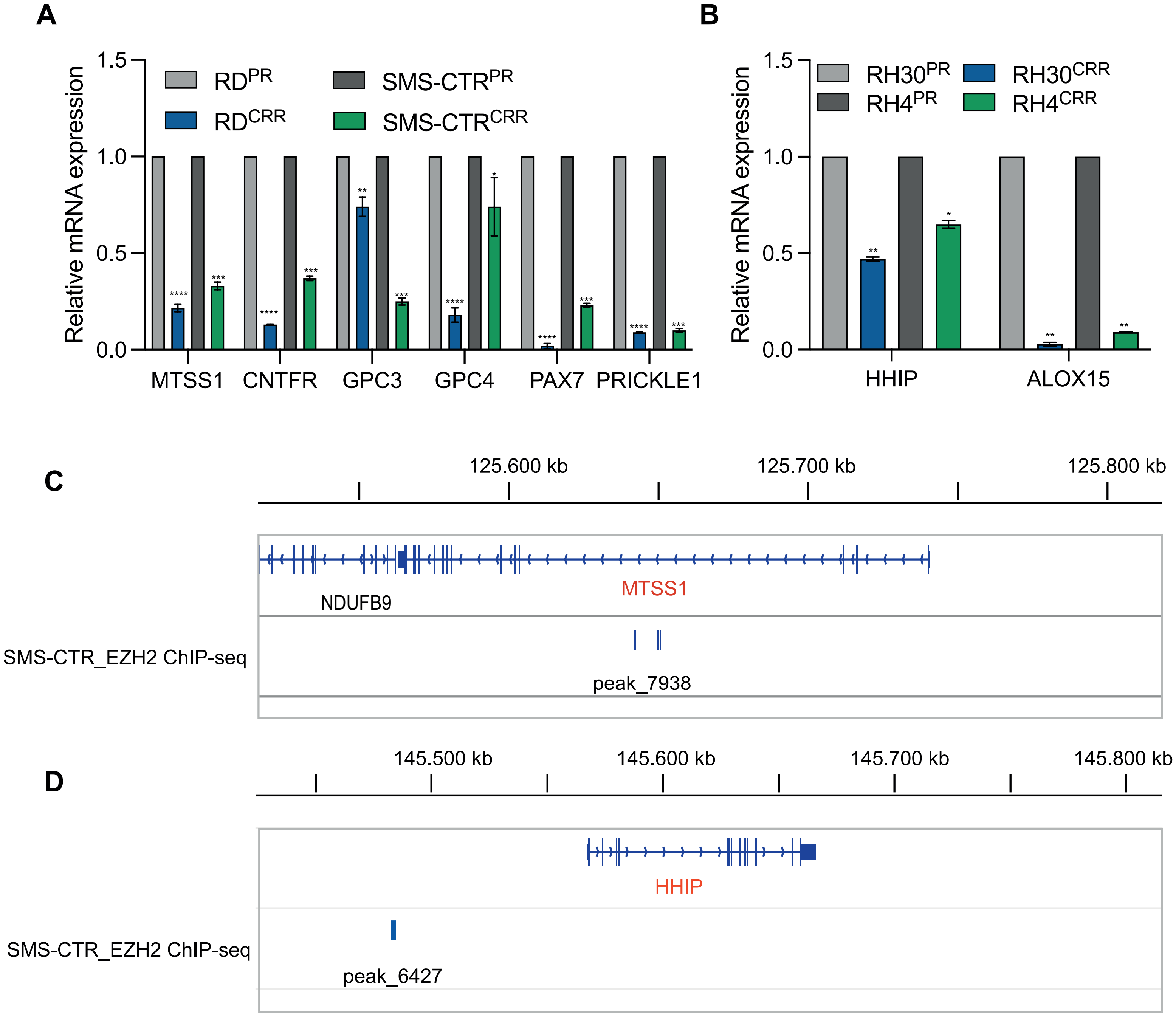

Supplementary Figure 2. MTSS1 and HHIP are directly regulated by EZH2. A) Histograms depicting fold change expression by qRT-PCR of MTSS1, CNTFR, GPC3, GPC4, PAX7, and PRICKLE1 in RD^{PR}, RD^{CRR}, SMS-CTR^{PR} and SMS-CTR^{CRR}. n = 3 independent experiments, data presented as mean values ± SD, one-way ANOVA. **B)** Histograms depicting fold change expression by qRT-PCR of HHIP and ALOX15 in $RH30^{PR}$, $RH30^{CRR}$, $RH4^{PR}$ and $RH4^{CRR}$. n = 3 independent experiments, data presented as mean values ± SD, one-way ANOVA. **C)** EZH2 ChIP-seq track in SMS-CTR cells from GSE85171 depicting peak_7938 on MTSS1 gene. **D)** EZH2 ChIP-seq track in SMS-CTR cells from GSE85171 depicting peak_6427 on HHIP gene. * $p<0.05$; ** $p<0.01$; *** $p<0.005$; **** $p<0.001$.

Supplementary Figure 3

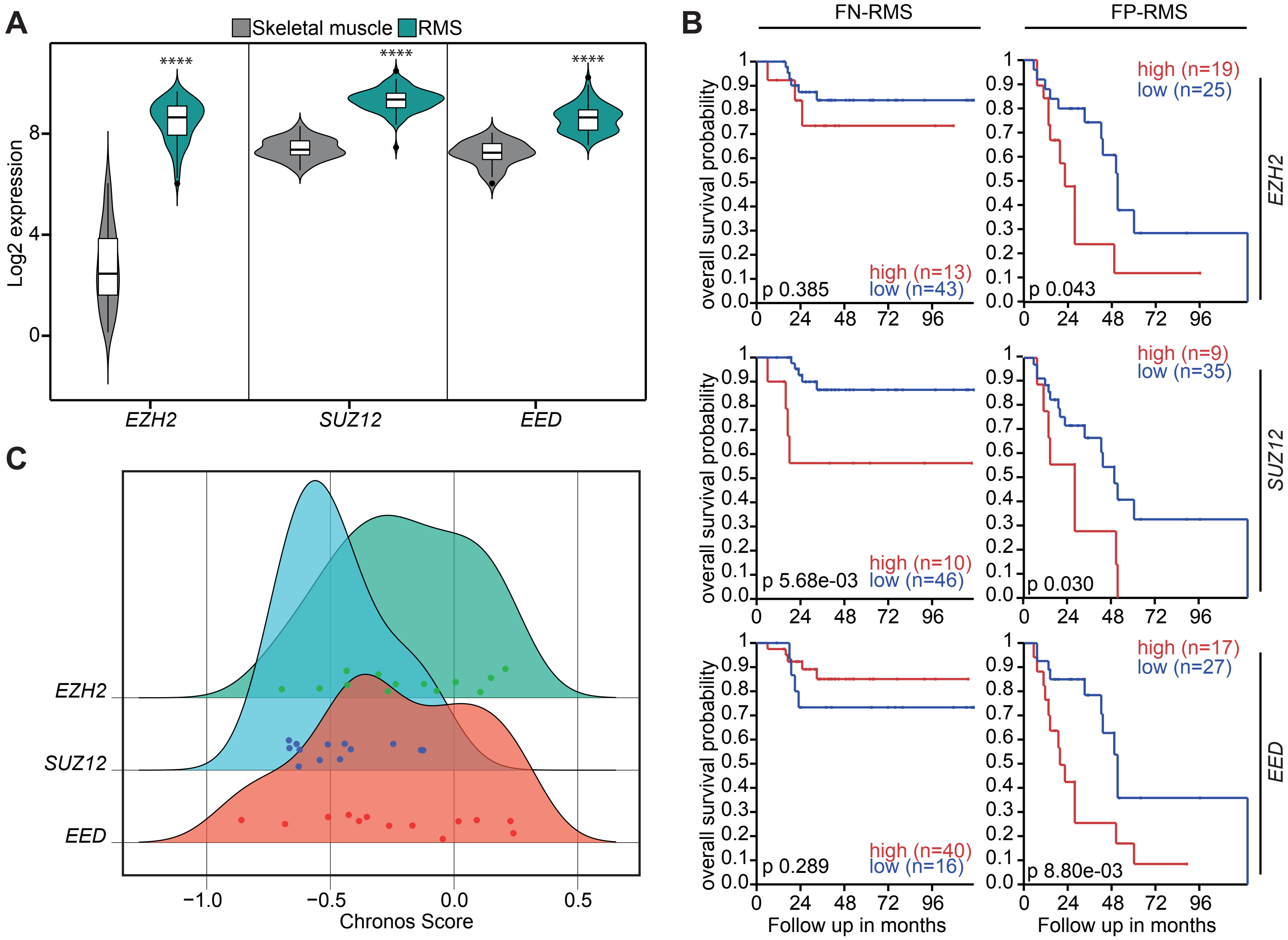

Supplementary Figure 3. PRC2 complex components are upregulated in RMS patients and their expression correlates with prognosis. A) Violin plots depicting EZH2, SUZ12 and EED expression levels among normal skeletal muscle tissue (n=40, GSE9103) and RMS patients (n=101, E-TABM-1202). **B)** Kaplan–Meyer plots depicting correlation between EZH2, SUZ12 and EED expression level (FN- (n = 57) and FP-RMS (n = 45) E-TABM-1202) patients' survival. **C)** Ridgeline plots depicting effects of EZH2, SUZ12 and EED depletion on RMS cancer cell lines obtained by DepMap (www.depmap.org). Dependency is reported as Chronos score.

Supplementary Figure 4

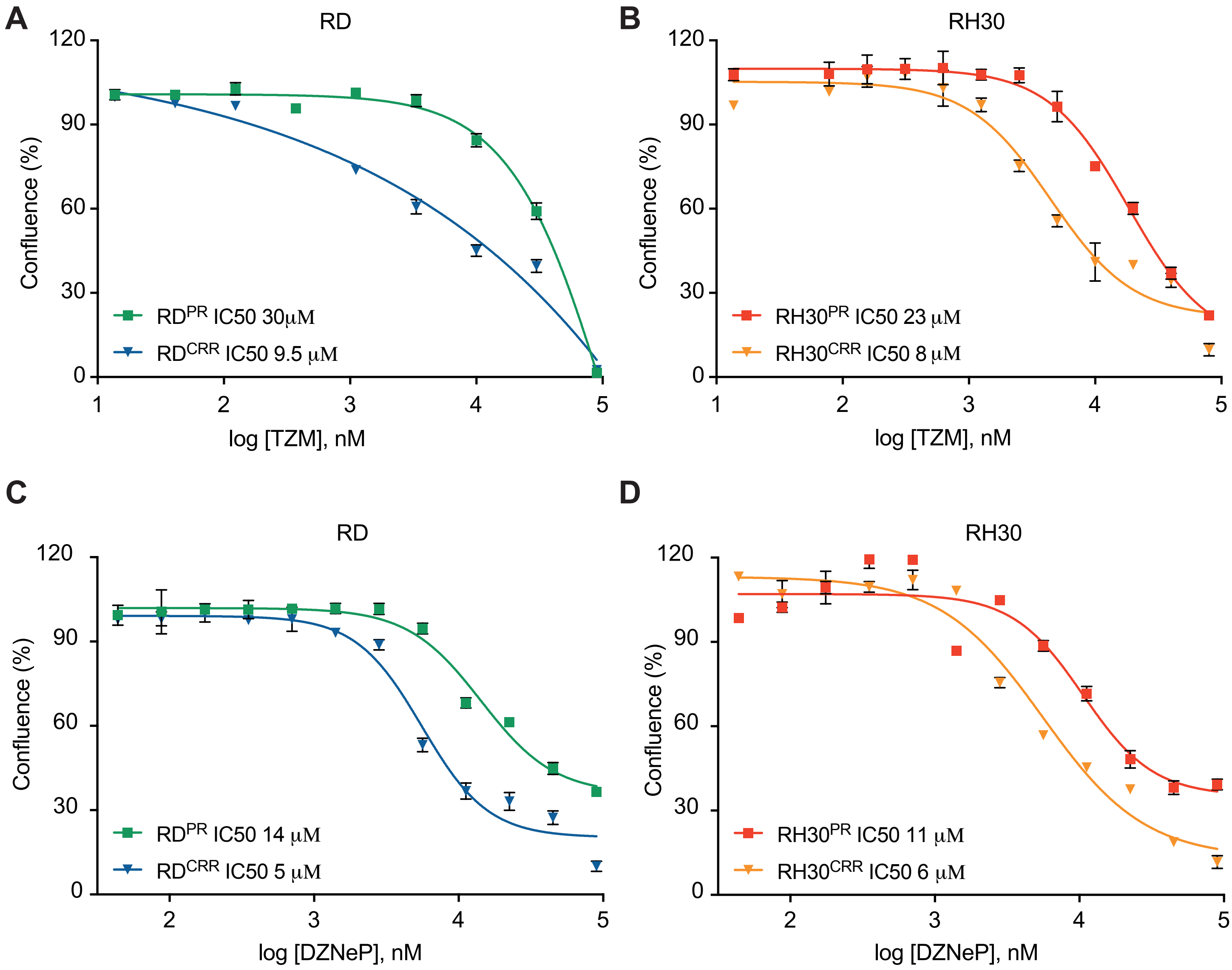

Supplementary Figure 4. EZH2 inhibition counteracts intrinsic and acquired radioresistance in vitro. A) and **B)** Dose-response curves of RMS^{PR} and RMS^{CRR} cells treated with increasing concentration of TZM. Graphs represent the mean of three independent experiments ± SEM. **C)** and **D)** Dose-response curves of RMS^{PR} and RMS^{CRR} cells treated with increasing concentration of DZNeP. Graphs represent the mean of three independent experiments ± SEM.

Supplementary Figure 5

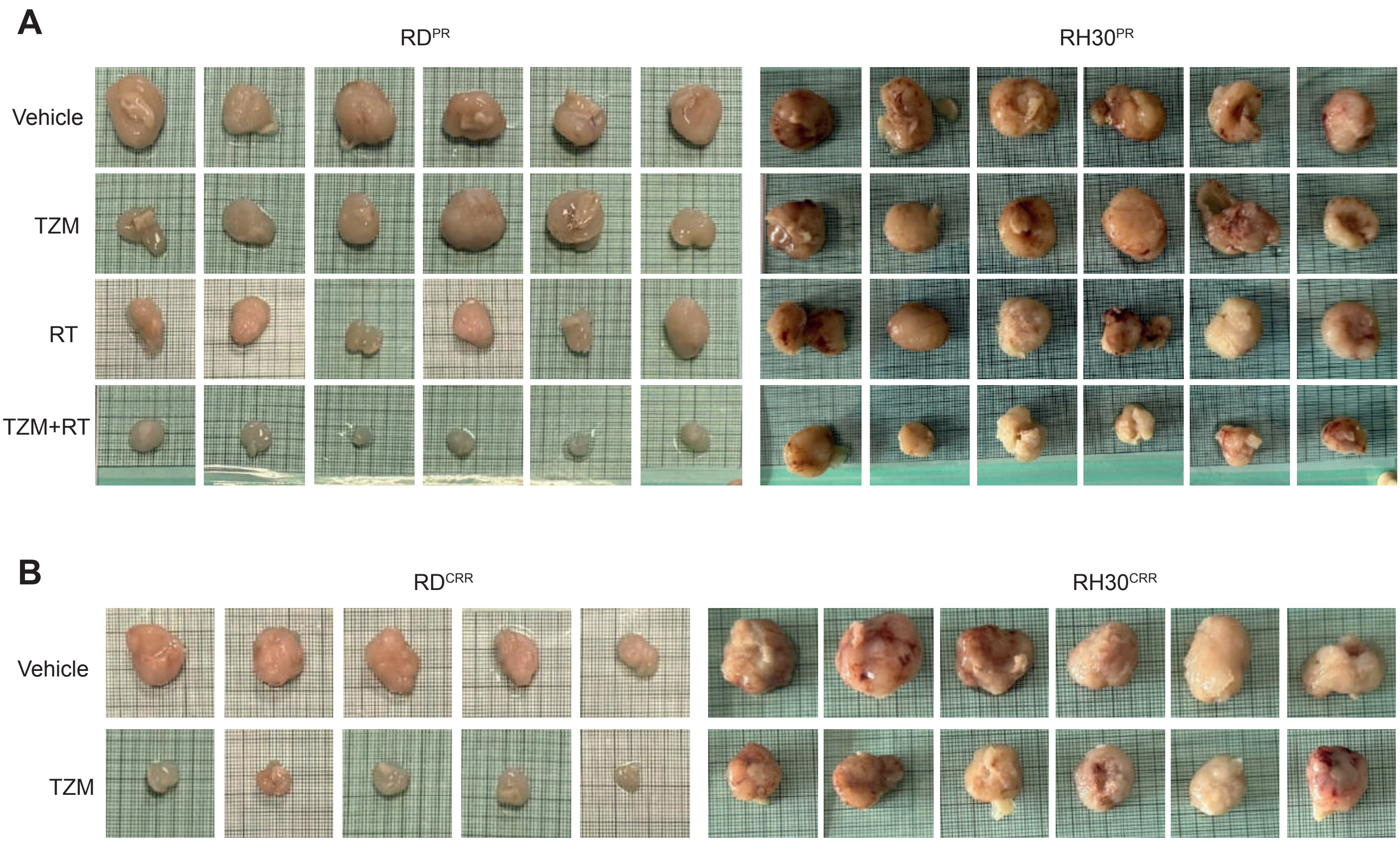

Supplementary Figure 5. TZM sensitizes RMS^{PR} cells to radiotherapy in vivo and counteracts RMS^{CRR} in vivo growth. A) Images of RD^{PR} and RH30^{PR} tumors explanted from mice pre-treated for 5 days with 75 mg/Kg of TZM and then irradiated each 2 days for 3 times with a single dose of 2 Gy of RT. **B)** Images of RD^{CRR} and RH30^{CRR} tumors explanted from mice treated with 75 mg/Kg of TZM.
