# Supplementary material for: Multi-omics analyses identify EZH2 as a central driver in rhabdomyosarcoma radioresistance and highlight Tazemetostat as an effective radiosensitizer in vitro and in vivo
Source: Cell Death Dis. 2026 Jun 1;17(1):683. doi: 10.1038/s41419-026-08938-0 (PMC13439469; doi:10.1038/s41419-026-08938-0)
Supplement: Supplementary file 2 [file 41419_2026_8938_MOESM2_ESM.pdf]

FIGURE 2A

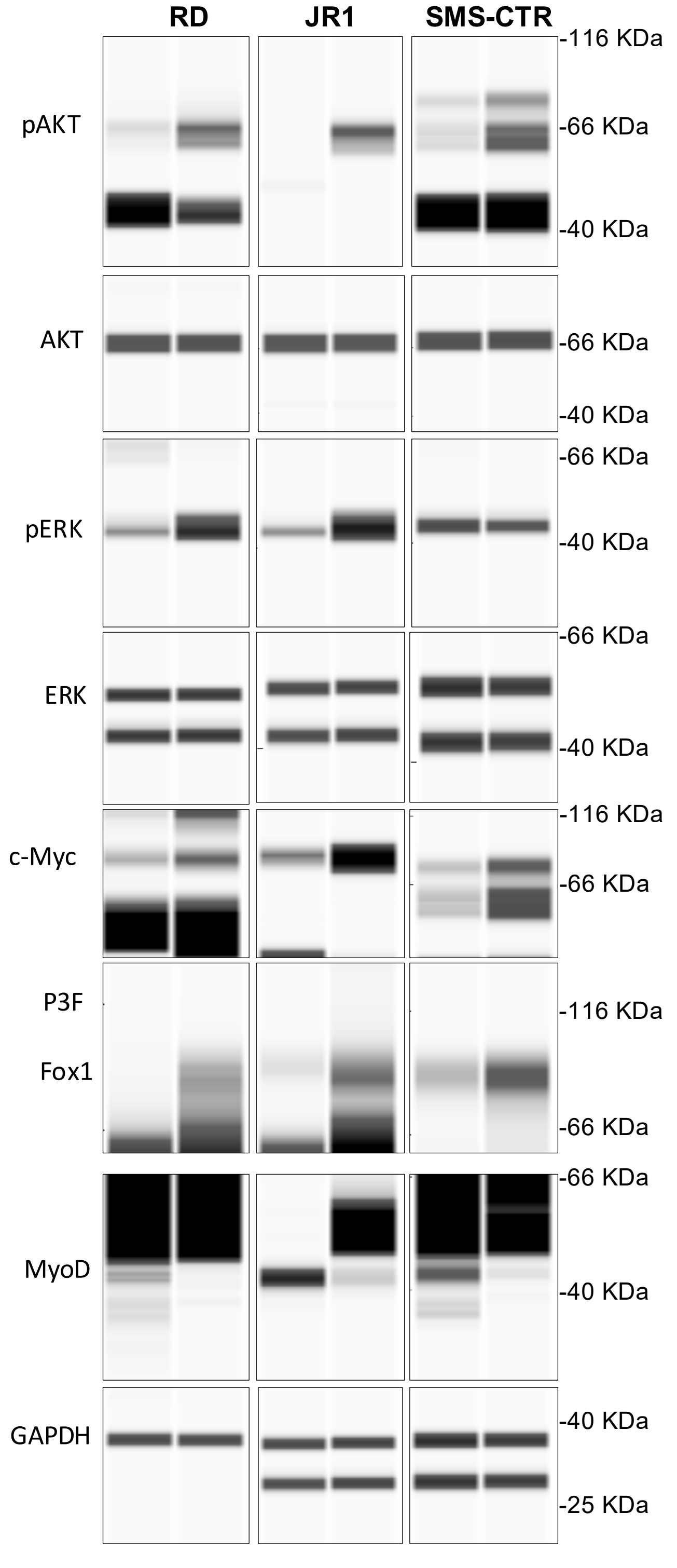

RD
JR1
SMS-CTR
pAKT
AKT
pERK
ERK
c-Myc
P3F
Fox1
MyoD
GAPDH
-116 KDa
-66 KDa
-40 KDa
-66 KDa
-40 KDa
-66 KDa
-40 KDa
-66 KDa
-40 KDa
-116 KDa
-66 KDa
-116 KDa
-66 KDa
-66 KDa
-40 KDa
-40 KDa
-25 KDa

Figure 2B

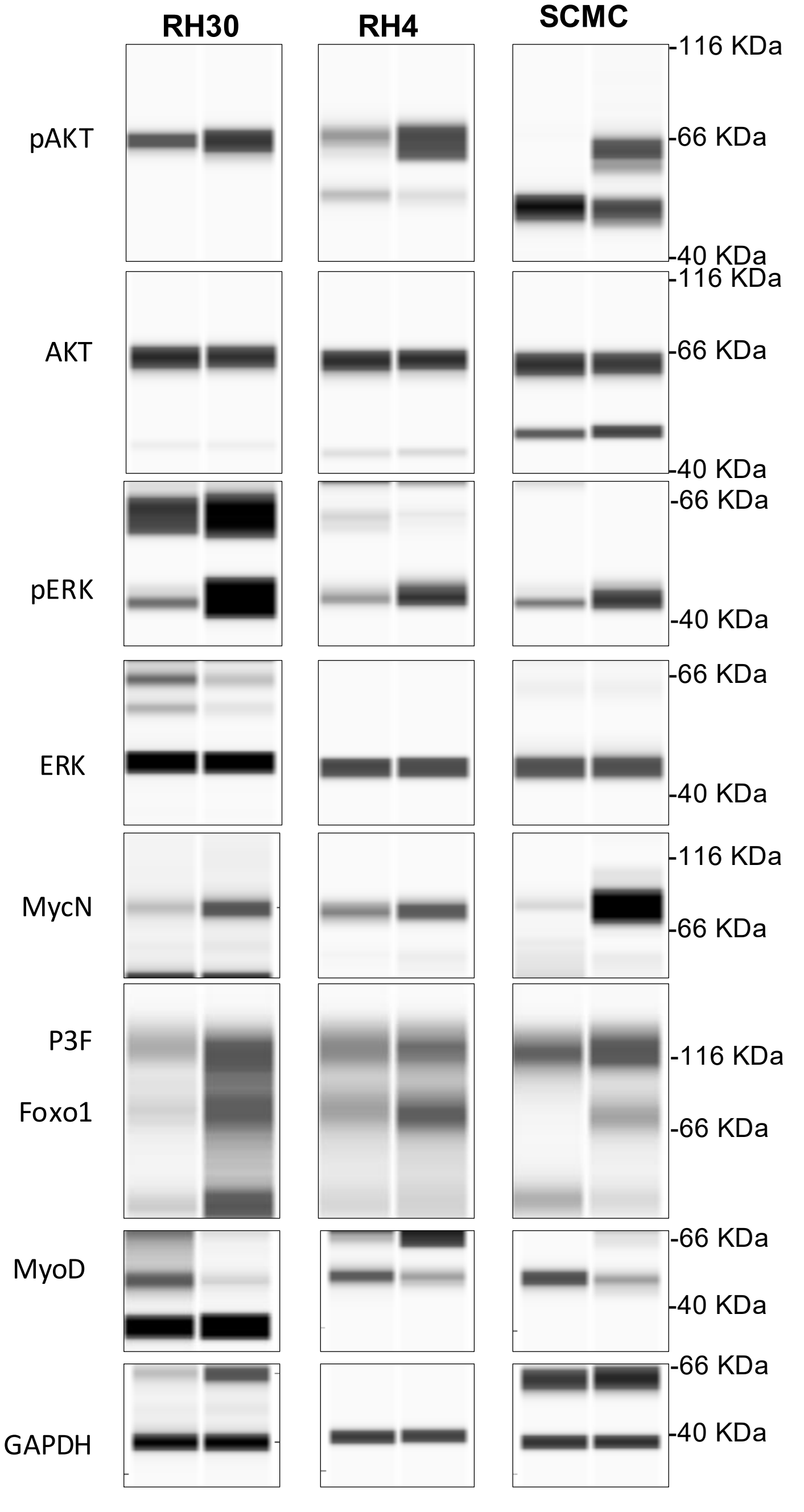

RH30
RH4
SCMC
pAKT
AKT
pERK
ERK
MycN
P3F
Foxo1
MyoD
GAPDH
-116 KDa
-66 KDa
-40 KDa
-116 KDa
-66 KDa
-40 KDa
-66 KDa
-40 KDa
-66 KDa
-40 KDa
-116 KDa
-66 KDa
-116 KDa
-66 KDa
-66 KDa
-40 KDa
-66 KDa
-40 KDa

FIGURE 4A

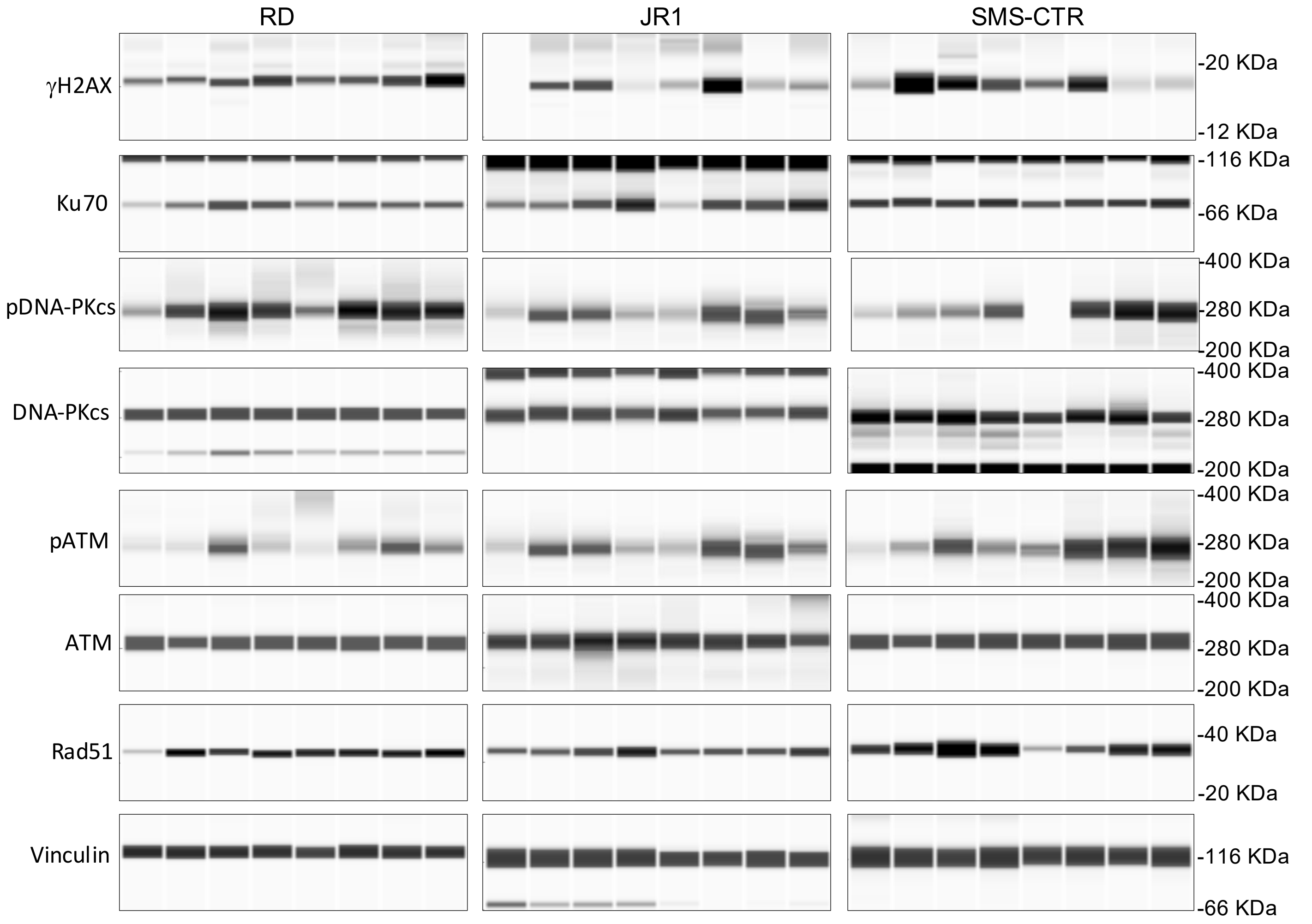
RD
JR1
SMS-CTR
γH2AX
Ku70
pDNA-PKcs
DNA-PKcs
pATM
ATM
Rad51
Vinculin
-20 KDa
-12 KDa
-116 KDa
-66 KDa
-400 KDa
-280 KDa
-200 KDa
-400 KDa
-280 KDa
-200 KDa
-400 KDa
-280 KDa
-200 KDa
-400 KDa
-280 KDa
-200 KDa
-40 KDa
-20 KDa
-116 KDa
-66 KDa

FIGURE 4B

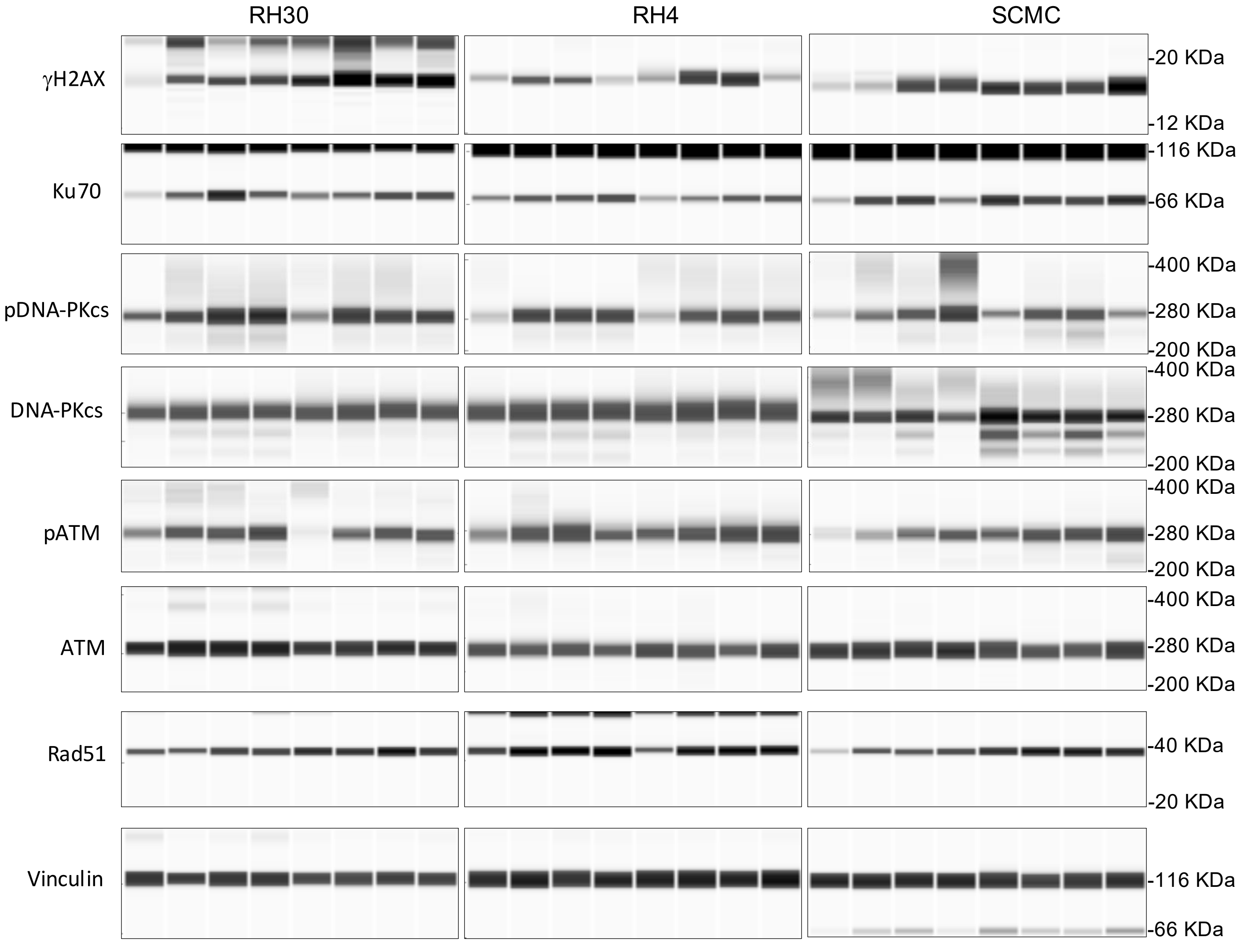

RH30
RH4
SCMC
γH2AX
Ku70
pDNA-PKcs
DNA-PKcs
pATM
ATM
Rad51
Vinculin
20 KDa
12 KDa
116 KDa
66 KDa
400 KDa
280 KDa
200 KDa
400 KDa
280 KDa
200 KDa
400 KDa
280 KDa
200 KDa
400 KDa
280 KDa
200 KDa
40 KDa
20 KDa
116 KDa
66 KDa

FIGURE 6

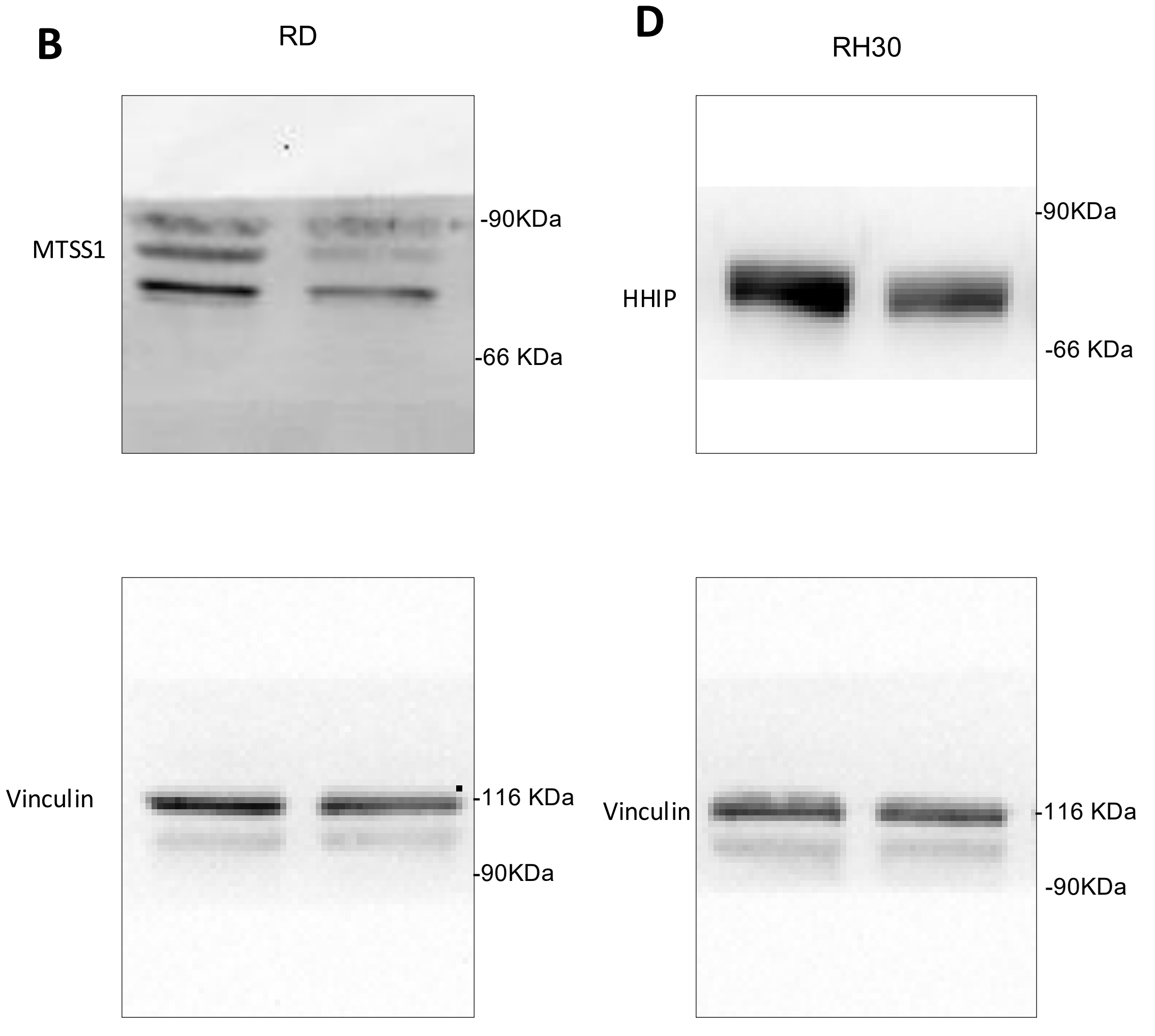
B
RD
MTSS1
-90KDa
-66 KDa
Vinculin
-116 KDa
-90KDa
D
RH30
HHIP
-90KDa
-66 KDa
Vinculin
-116 KDa
-90KDa

FIGURE 6

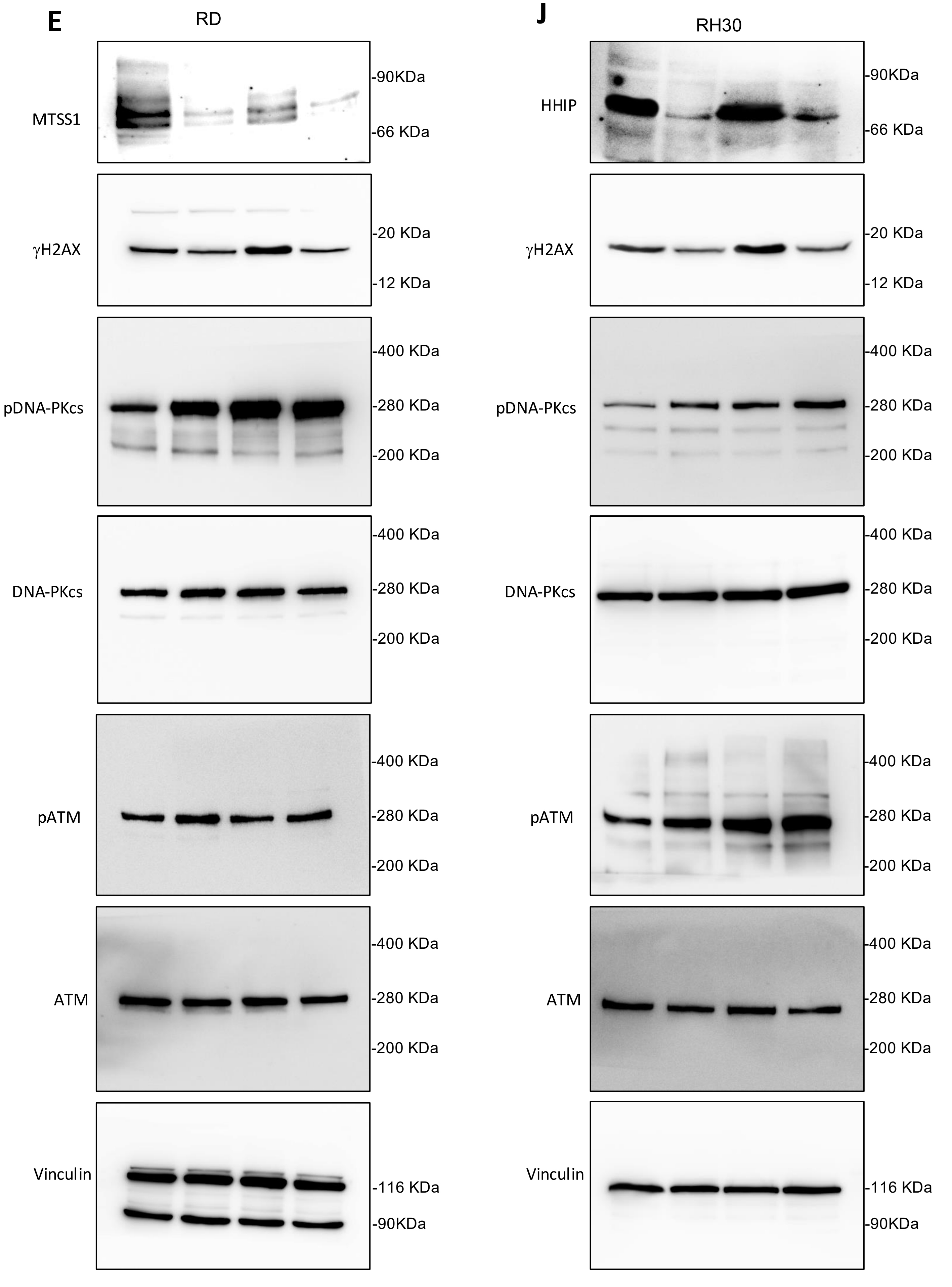
E
RD
MTSS1
-90KDa
-66 KDa
γH2AX
-20 KDa
-12 KDa
pDNA-PKcs
-400 KDa
-280 KDa
-200 KDa
DNA-PKcs
-400 KDa
-280 KDa
-200 KDa
pATM
-400 KDa
-280 KDa
-200 KDa
ATM
-400 KDa
-280 KDa
-200 KDa
Vinculin
-116 KDa
-90KDa
J
RH30
HHIP
-90KDa
-66 KDa
γH2AX
-20 KDa
-12 KDa
pDNA-PKcs
-400 KDa
-280 KDa
-200 KDa
DNA-PKcs
-400 KDa
-280 KDa
-200 KDa
pATM
-400 KDa
-280 KDa
-200 KDa
ATM
-400 KDa
-280 KDa
-200 KDa
Vinculin
-116 KDa
-90KDa

FIGURE 7A

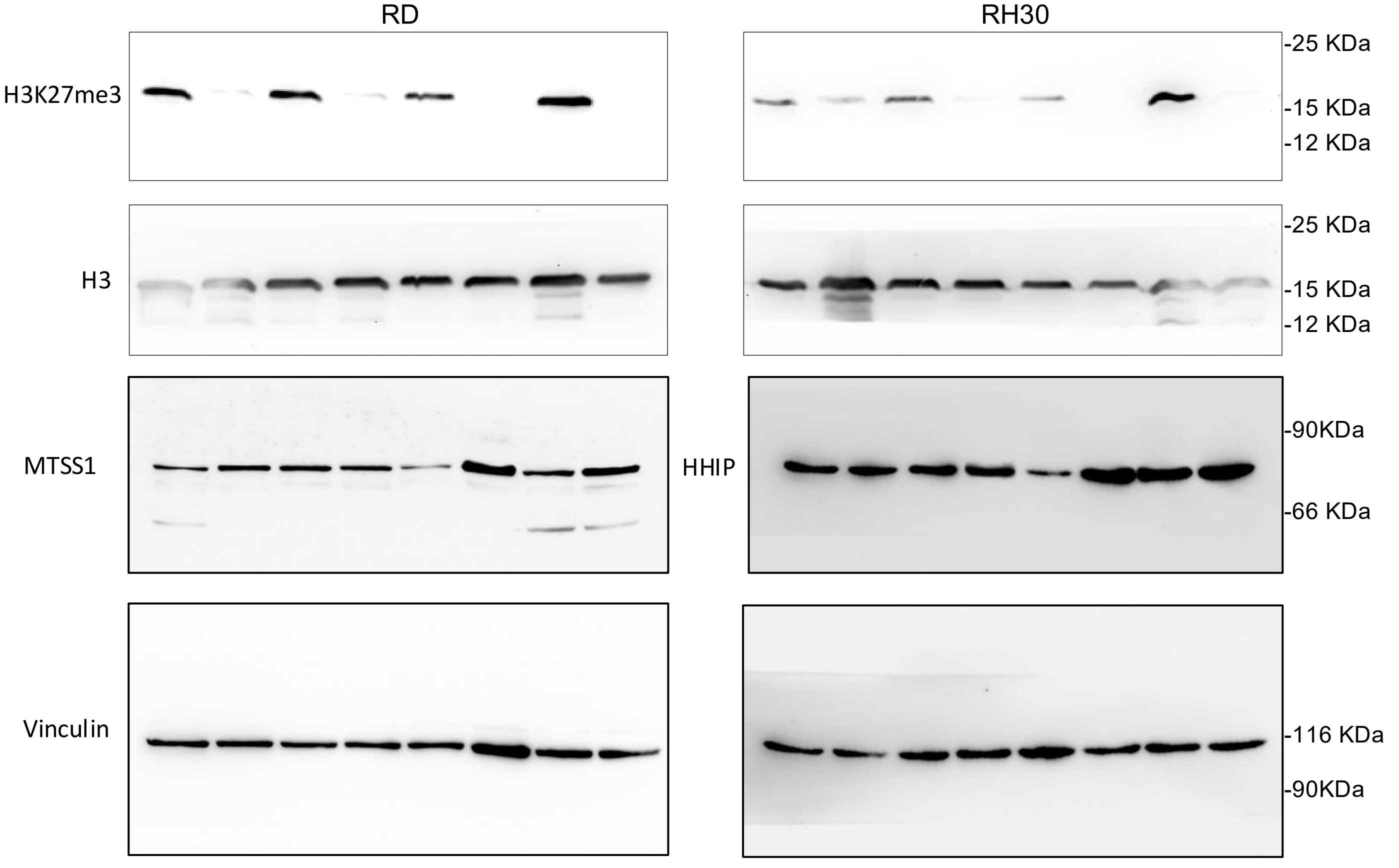
RD
RH30
H3K27me3
H3
MTSS1
HHIP
Vinculin
-25 KDa
-15 KDa
-12 KDa
-25 KDa
-15 KDa
-12 KDa
-90KDa
-66 KDa
-116 KDa
-90KDa

FIGURE 8A

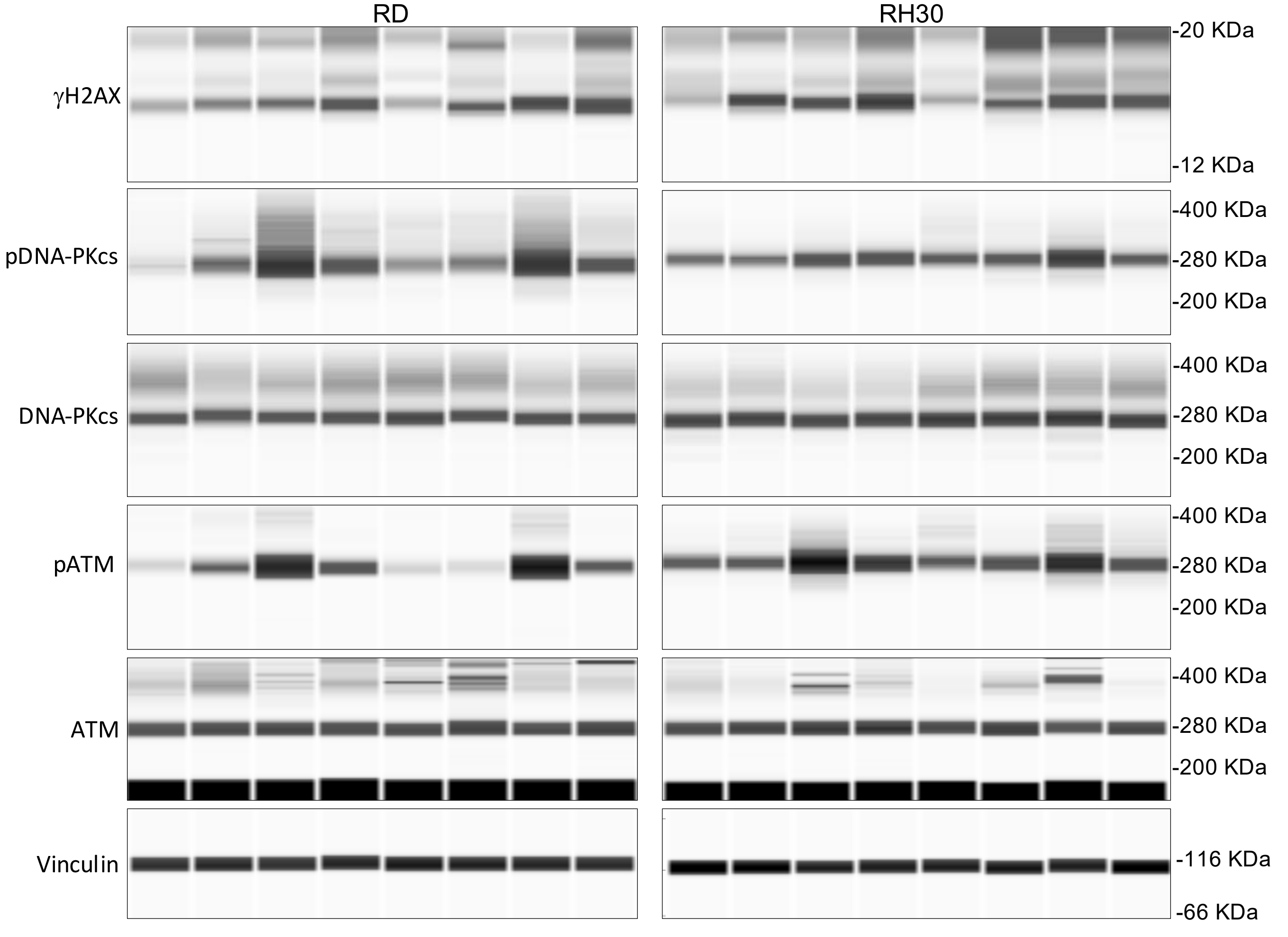

RD
RH30
γH2AX
pDNA-PKcs
DNA-PKcs
pATM
ATM
Vinculin
-20 KDa
-12 KDa
-400 KDa
-280 KDa
-200 KDa
-400 KDa
-280 KDa
-200 KDa
-400 KDa
-280 KDa
-200 KDa
-400 KDa
-280 KDa
-200 KDa
-116 KDa
-66 KDa
